# Supplementary material for: Shikonin Inhibits Cell Growth of Sunitinib-Resistant Renal Cell Carcinoma by Activating the Necrosome Complex and Inhibiting the AKT/mTOR Signaling Pathway
Source: Cancers (Basel). 2022 Feb 22;14(5):1114. doi: 10.3390/cancers14051114 (PMC8909272; doi:10.3390/cancers14051114)
Supplement: Supplementary file 1 [file cancers-14-01114-s001.zip › cancers-1513825-supplementary.pdf]

Cell cycle regulating proteins

p21, p27, Cyclin A, Cylin B, CDK1, pCDK1, CDK2, pCDK2

Figure S1

CAKI-1

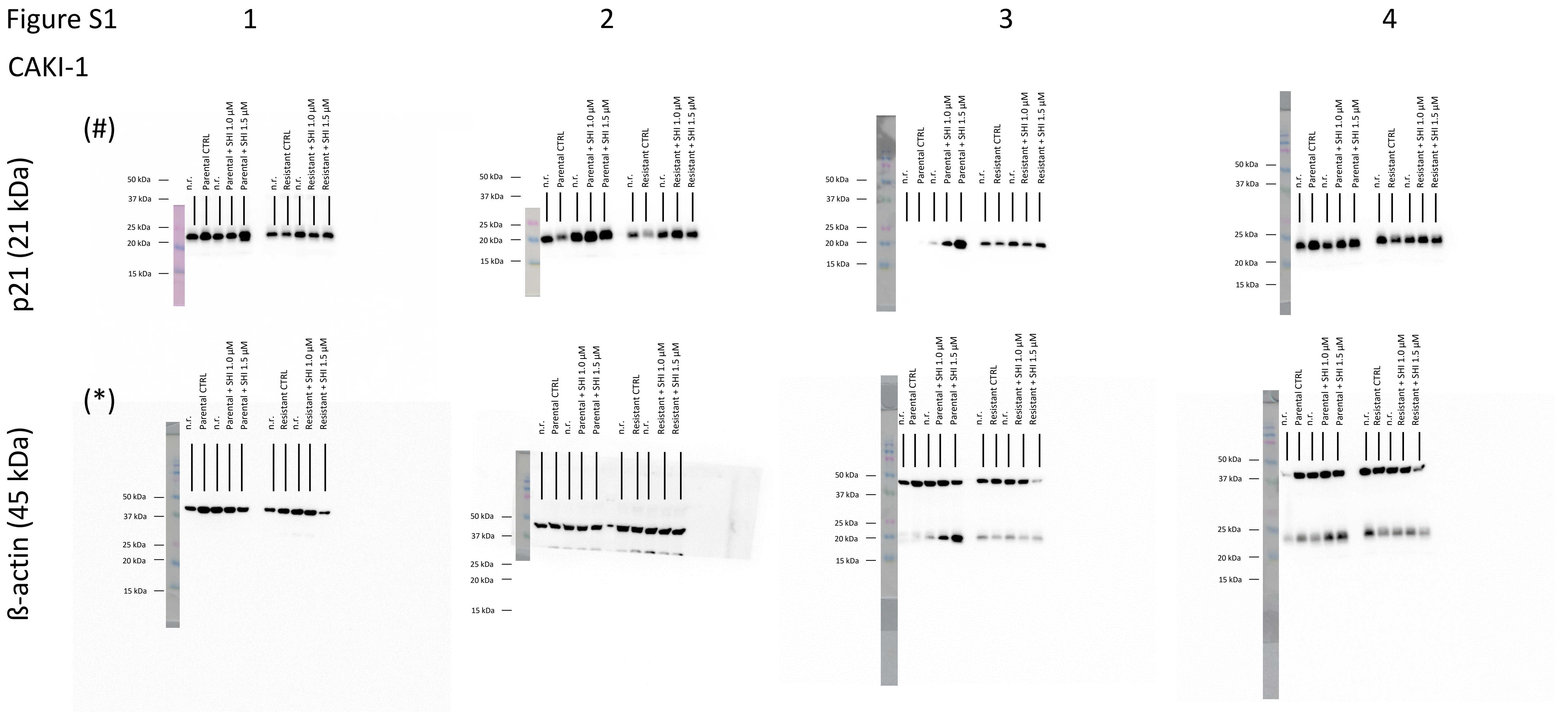

Figure S1a: Detailed information about Figure 4 - Protein expression and activity of cell cycle regulating proteins in parental and sunitinib-resistant Caki-1 cells after 48 h exposure to 1 or 1.5 µM SHI. Protein expression of p21 (#), corresponding protein expression of ß-actin (*). n.r. = not relevant.

Figure S1

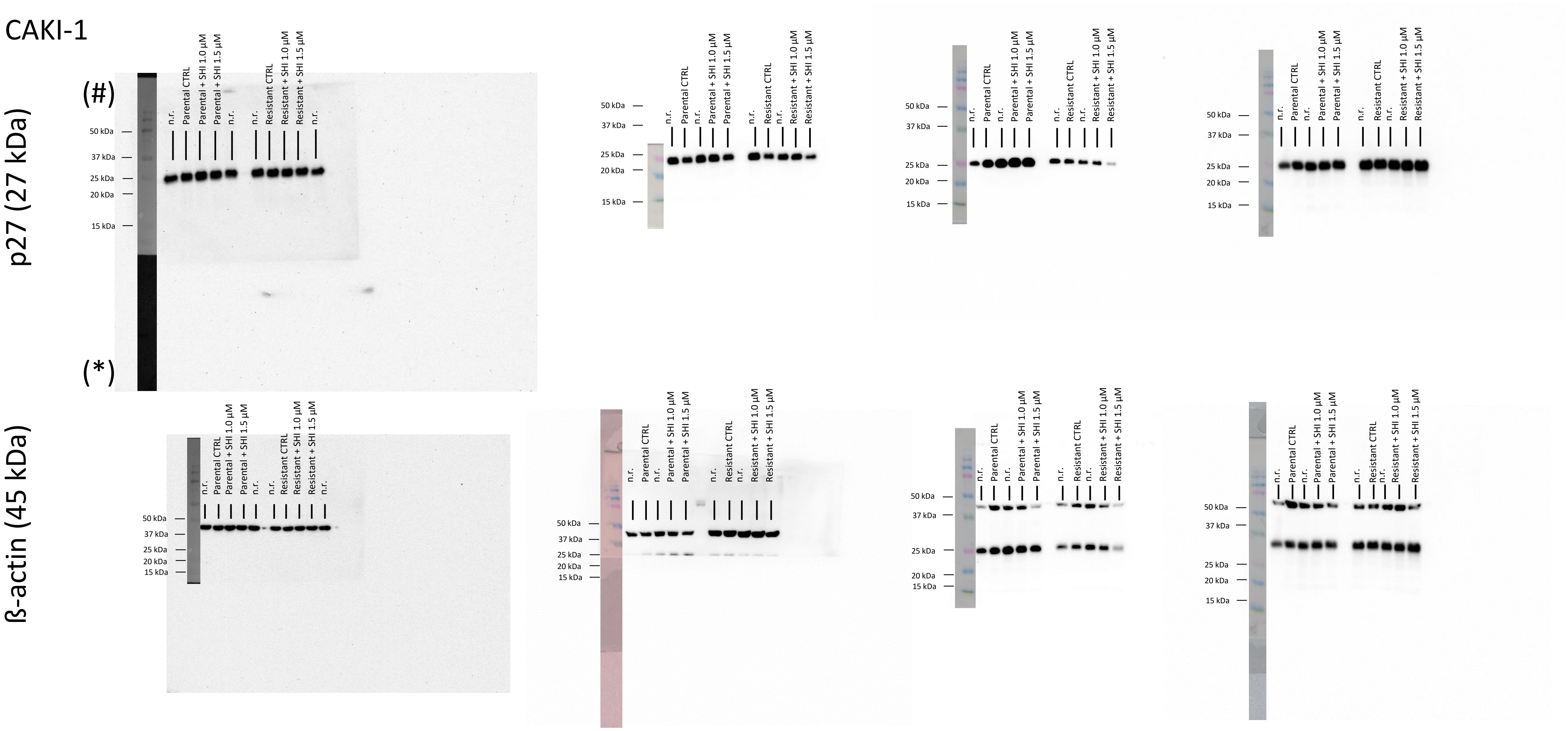

Figure S1b: Detailed information about Figure 4 - Protein expression and activity of cell cycle regulating proteins in parental and sunitinib-resistant Caki-1 cells after 48 h exposure to 1 or 1.5 µM SHI. Protein expression of p27 (#), corresponding protein expression of ß-actin (*). n.r. = not relevant.

Figure S1

CAKI-1

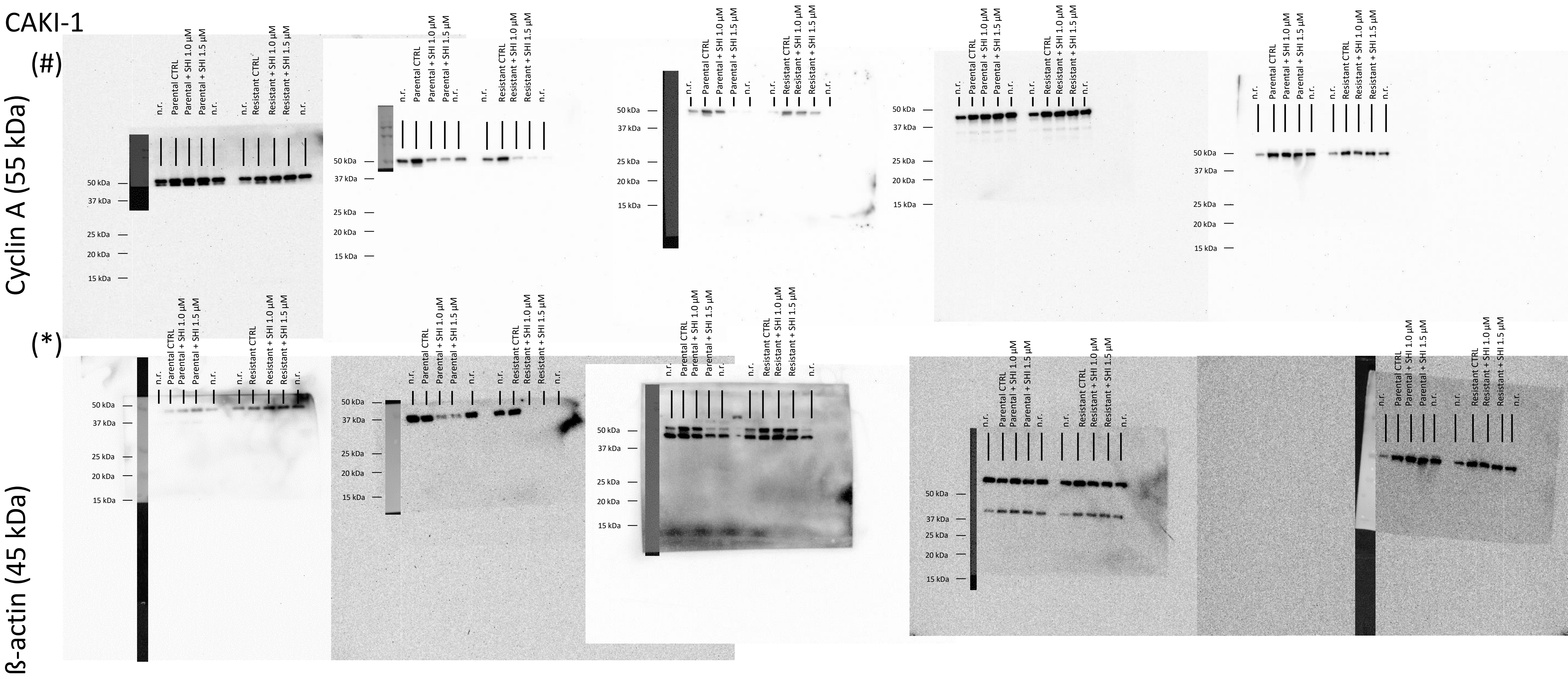

Figure S1c: Detailed information about Figure 4 - Protein expression and activity of cell cycle regulating proteins in parental and sunitinib-resistant Caki-1 cells after 48 h exposure to 1 or 1.5 µM SHI. Protein expression of Cyclin A (#), corresponding protein expression of ß-actin (*). n.r. = not relevant.

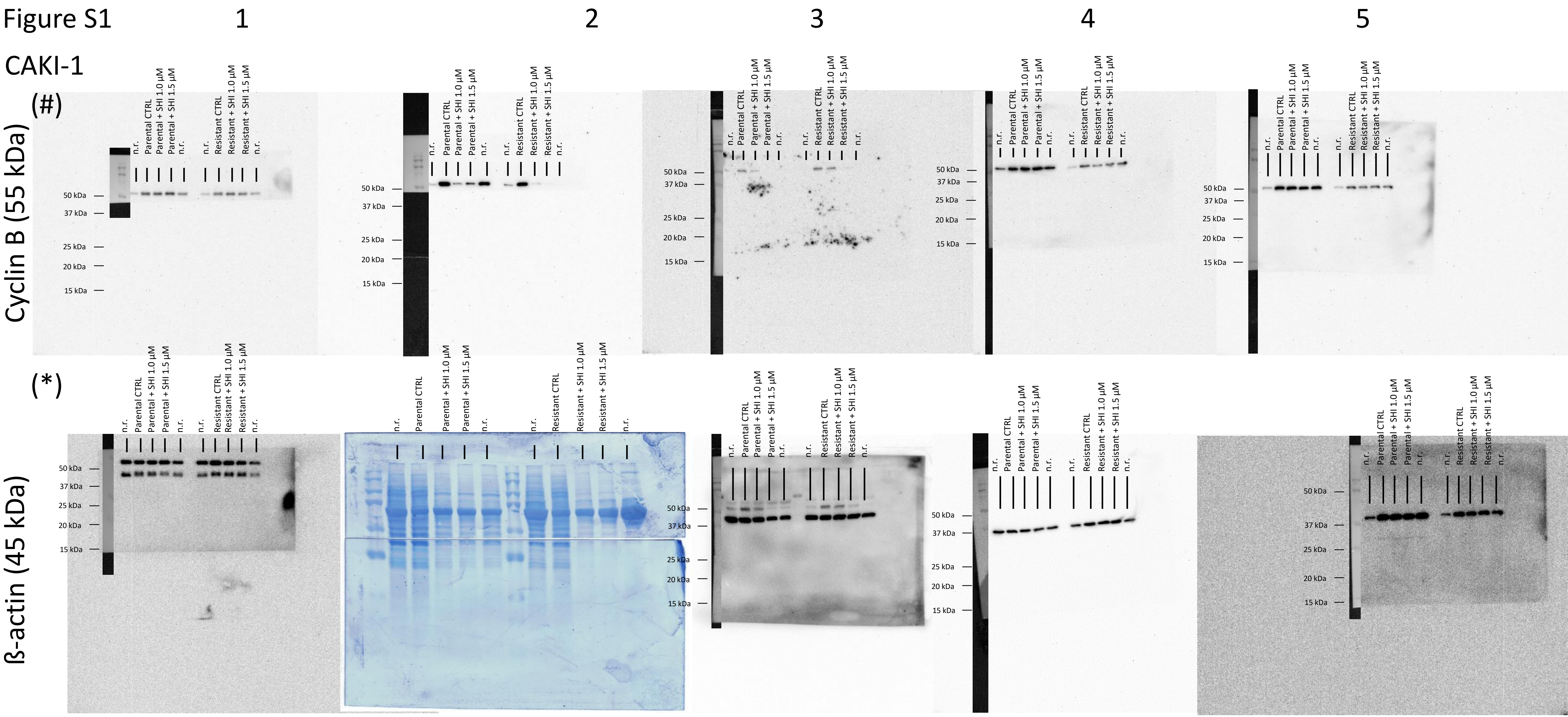

Figure S1d: Detailed information about Figure 4 - Protein expression and activity of cell cycle regulating proteins in parental and sunitinib-resistant Caki-1 cells after 48 h exposure to 1 or 1.5 µM SHI. Protein expression of Cyclin B (#), corresponding protein expression of ß-actin or Coomassie blue staining of total protein (*). n.r. = not relevant.

Figure S1

CAKI-1

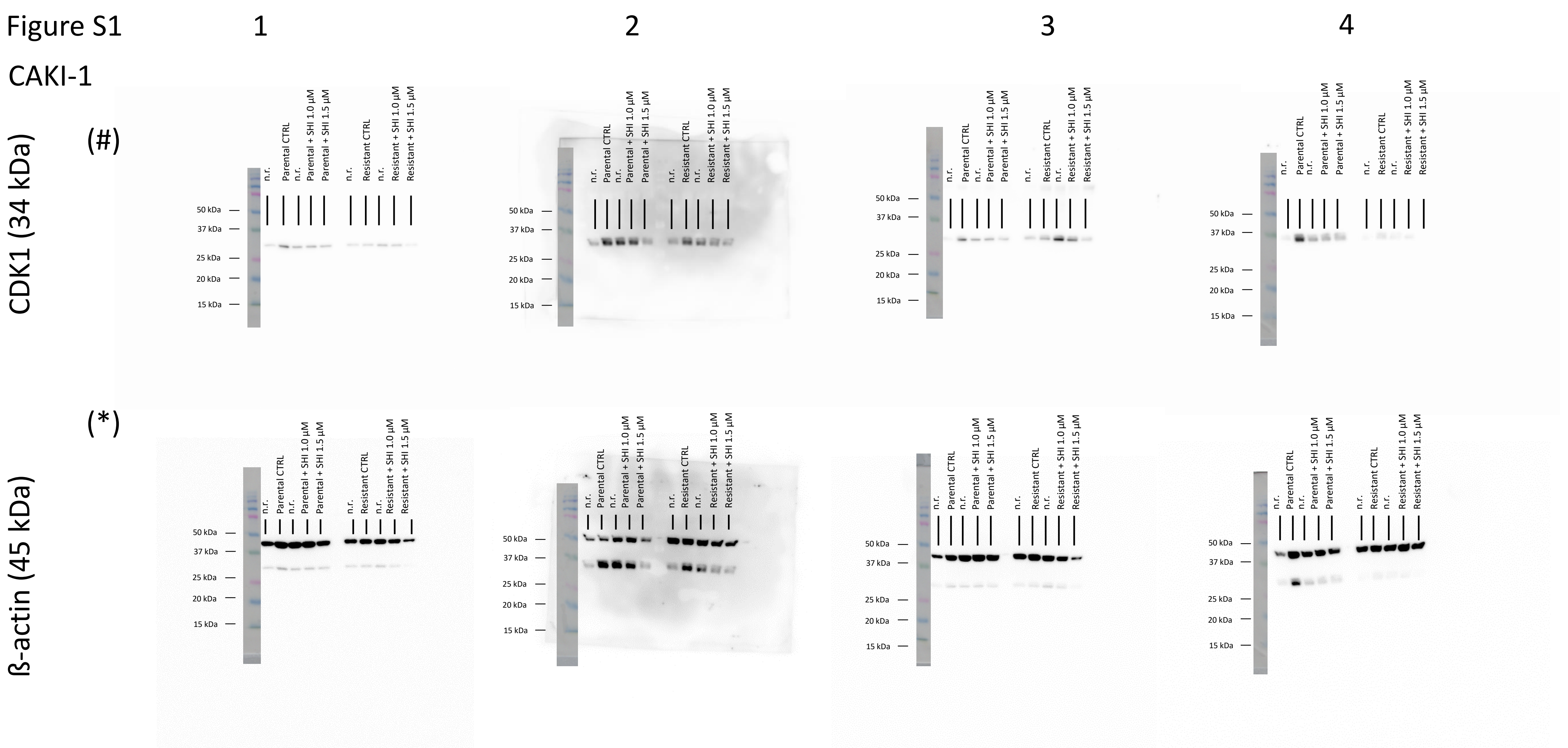

Figure S1e: Detailed information about Figure 4 - Protein expression and activity of cell cycle regulating proteins in parental and sunitinib-resistant Caki-1 cells after 48 h exposure to 1 or 1.5 µM SHI. Protein expression of CDK1 (#), corresponding protein expression of ß-actin (*). n.r. = not relevant.

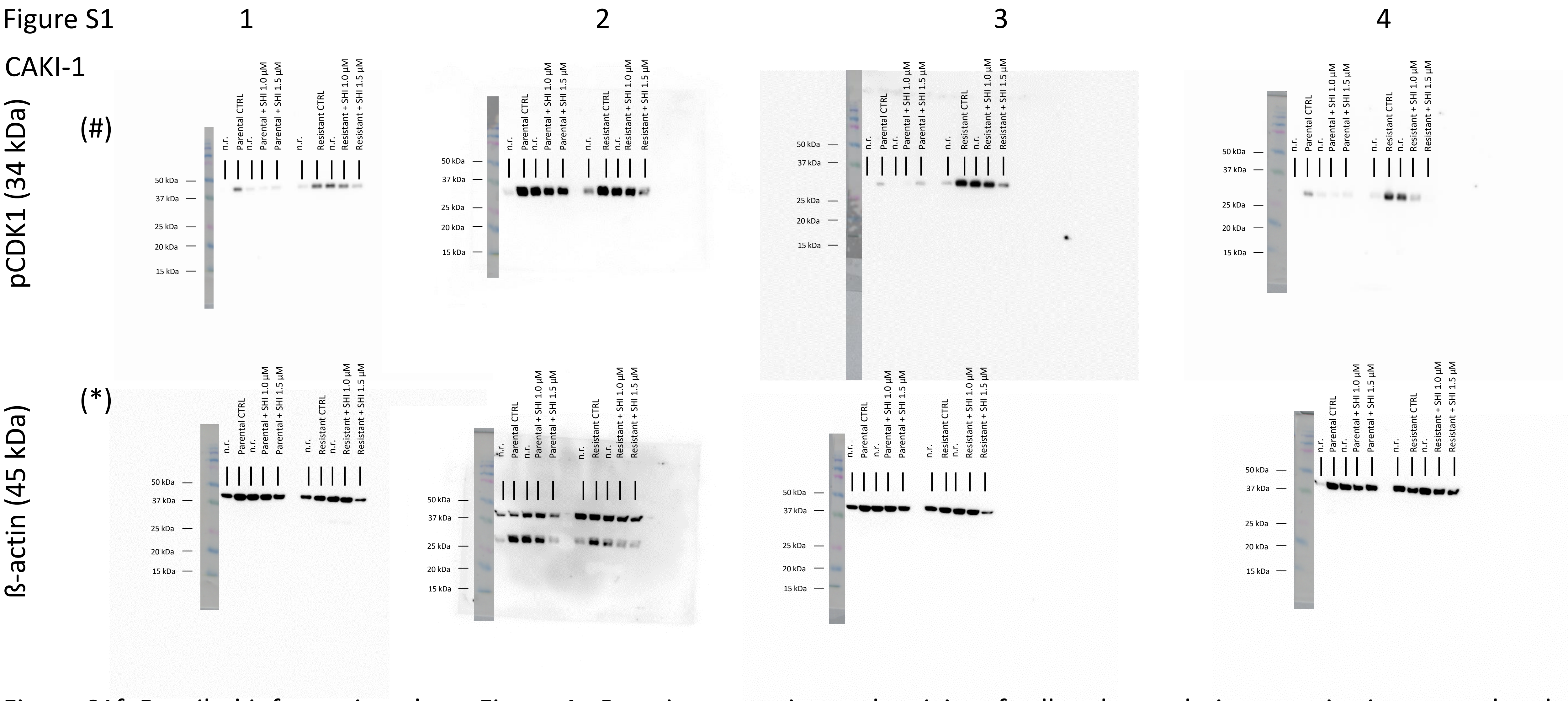

Figure S1f: Detailed information about Figure 4 - Protein expression and activity of cell cycle regulating proteins in parental and sunitinib-resistant Caki-1 cells after 48 h exposure to 1 or 1.5 µM SHI. Protein expression of pCDK1 (#), corresponding protein expression of ß-actin (*). n.r. = not relevant.

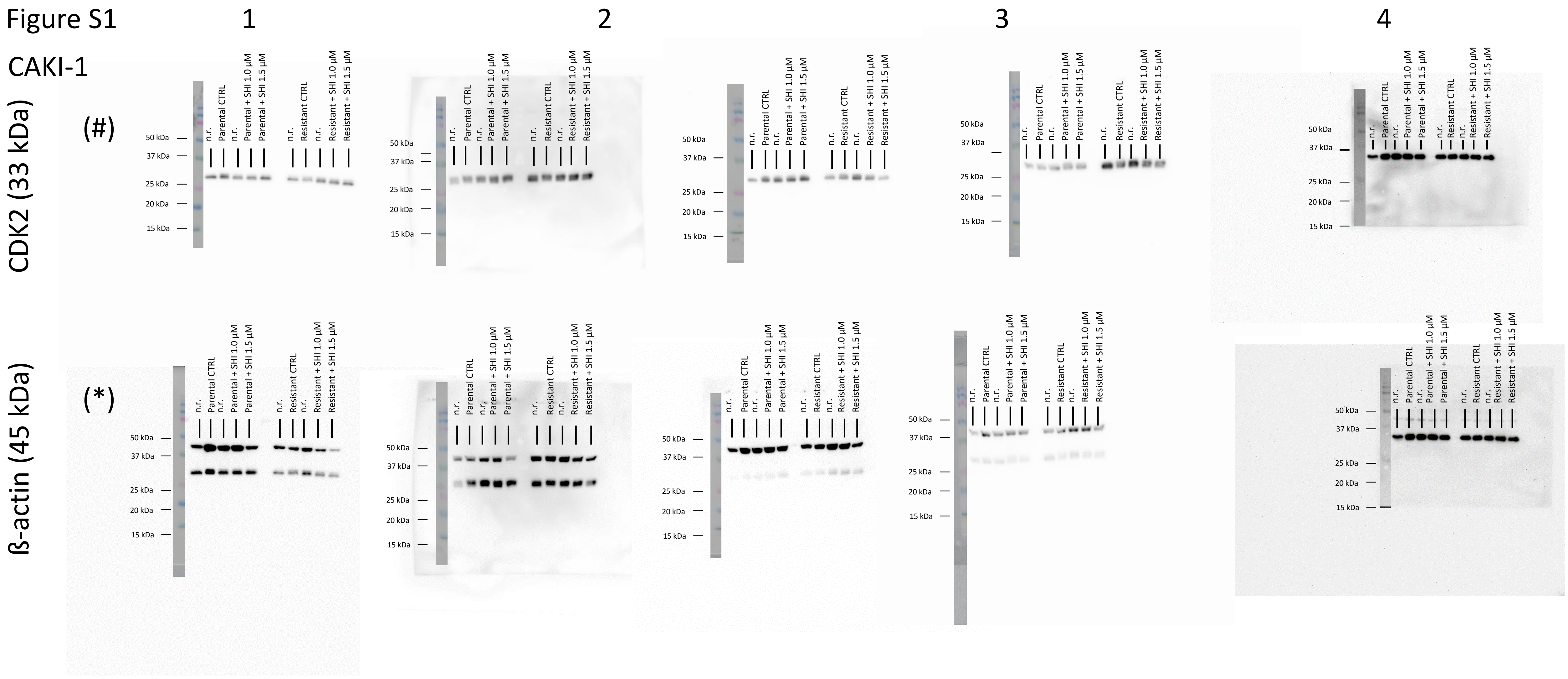

Figure S1g: Detailed information about Figure 4 - Protein expression and activity of cell cycle regulating proteins in parental and sunitinib-resistant Caki-1 cells after 48 h exposure to 1 or 1.5 µM SHI. Protein expression of CDK2 (#), corresponding protein expression of ß-actin (*). n.r. = not relevant.

Figure S2

786-O

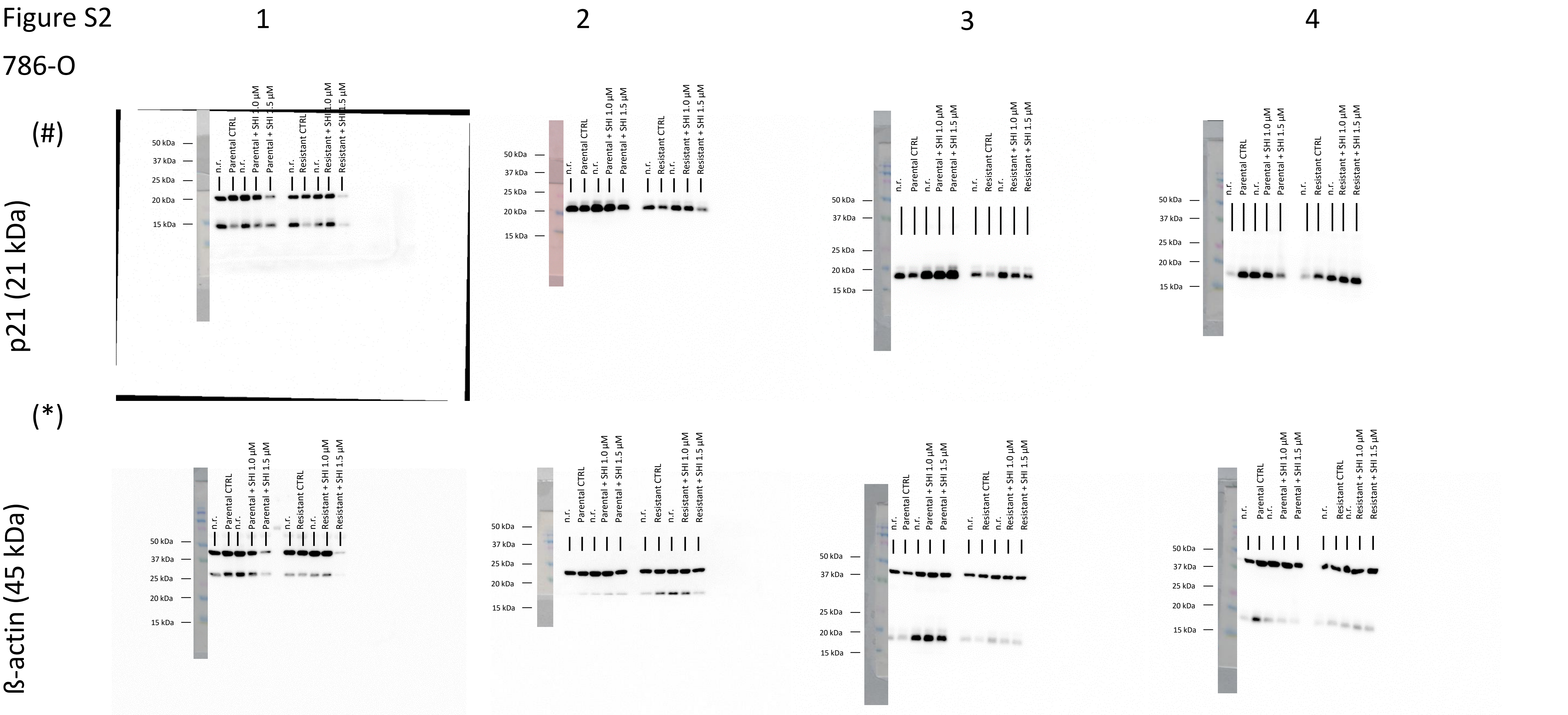

Figure S2a: Detailed information about Figure 5 - Protein expression and activity of cell cycle regulating proteins in parental and sunitinib-resistant 786-O cells after 48 h exposure to 1 or 1.5 µM SHI. Protein expression of p21 (#), corresponding protein expression of ß-actin (*). n.r. = not relevant.

Figure S2

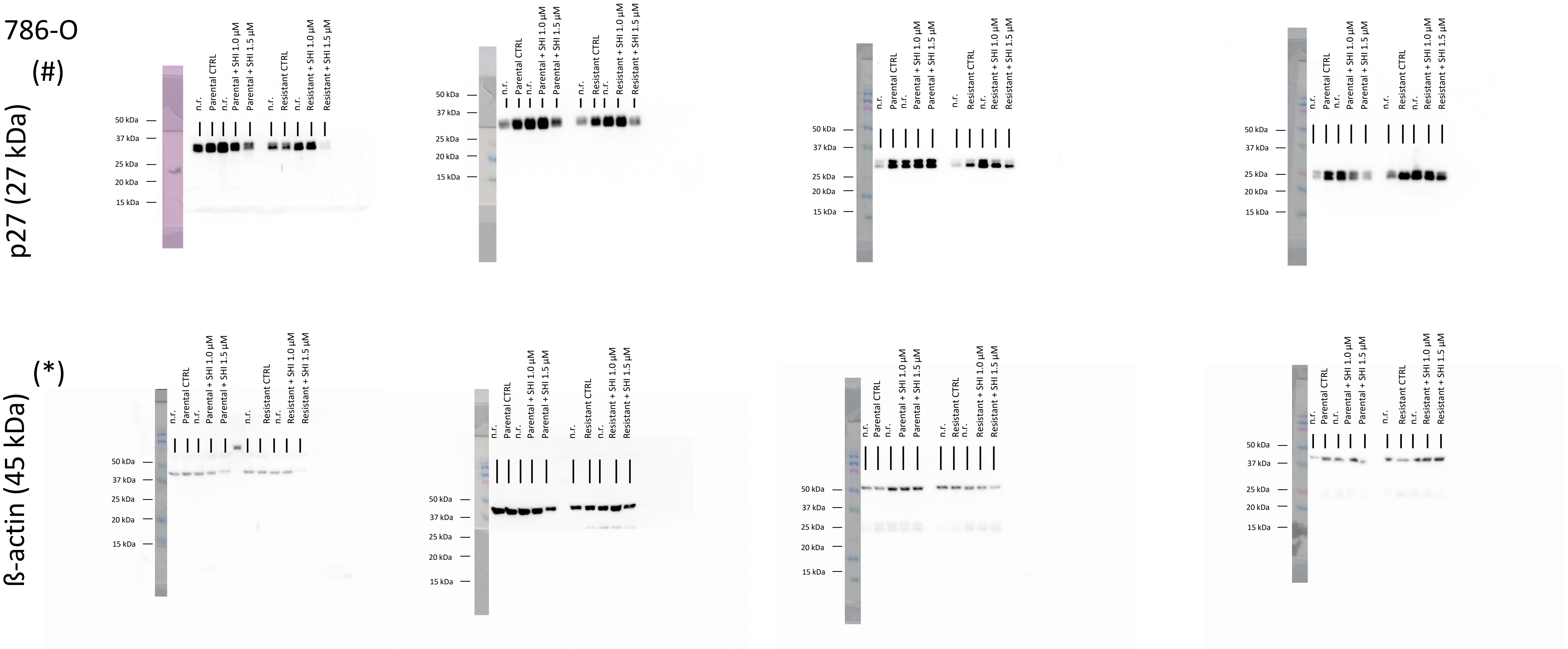

Figure S2b: Detailed information about Figure 5 - Protein expression and activity of cell cycle regulating proteins in parental and sunitinib-resistant 786-O cells after 48 h exposure to 1 or 1.5 µM SHI. Protein expression of p27 (#), corresponding protein expression of ß-actin (*). n.r. = not relevant.

Figure S2

786-O

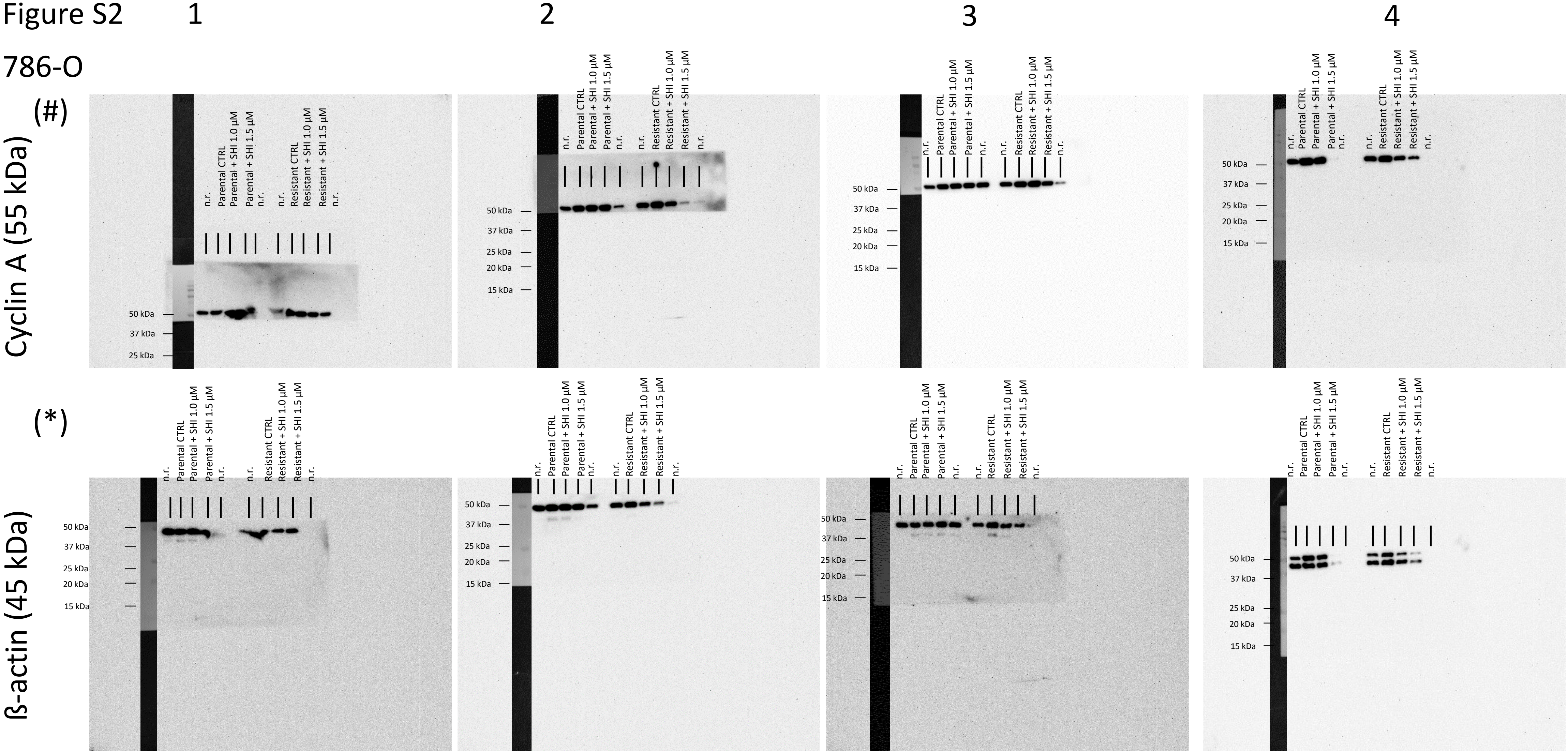

Figure S2c: Detailed information about Figure 5 - Protein expression and activity of cell cycle regulating proteins in parental and sunitinib-resistant 786-O cells after 48 h exposure to 1 or 1.5 µM SHI. Protein expression of Cyclin A (#), corresponding protein expression of ß-actin (*). n.r. = not relevant.

Figure S2

5 6

786-O

(#)

Cyclin A (55 kDa)

(*)

ß-actin (45 kDa)

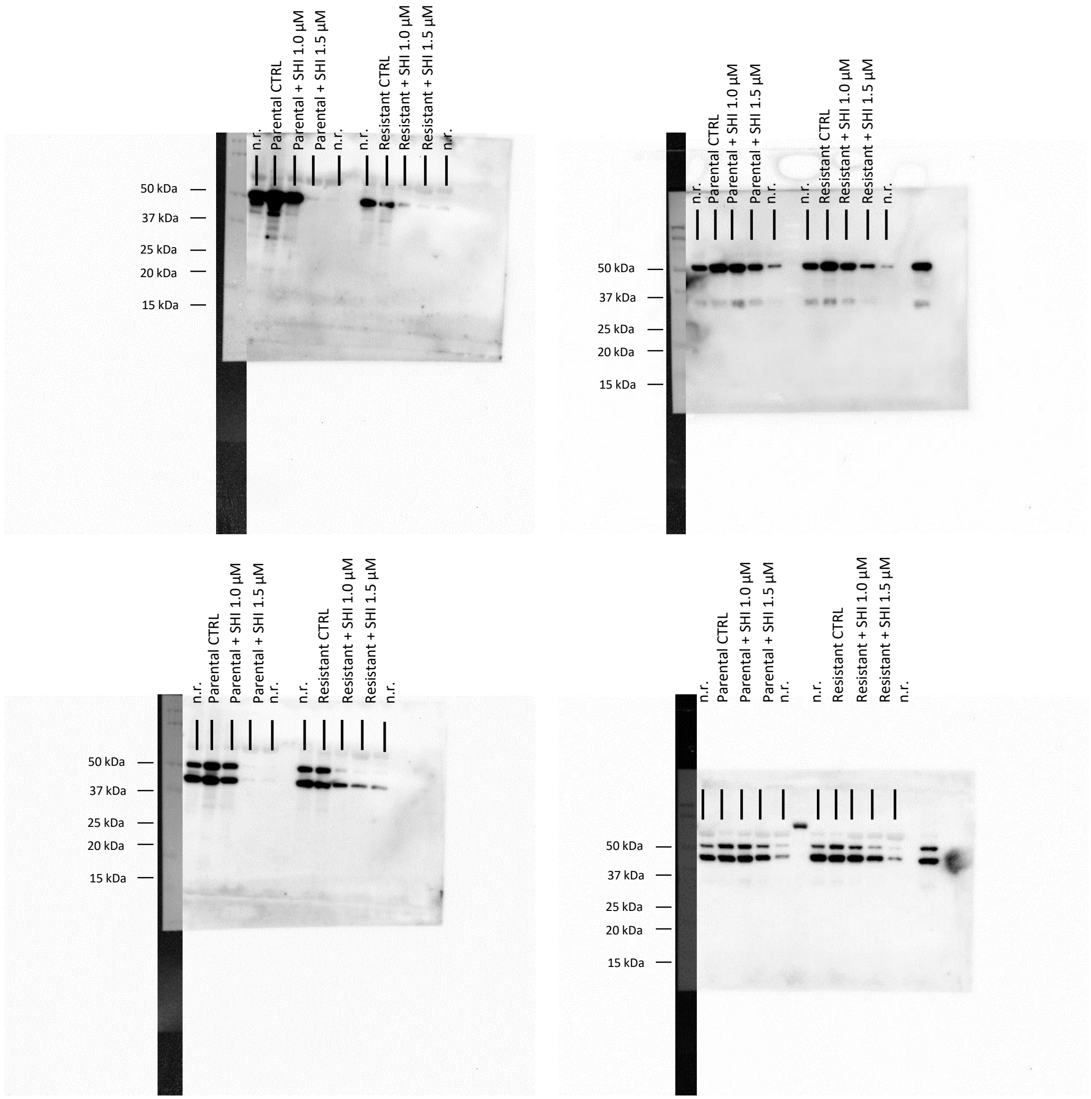

Figure S2c: Detailed information about Figure 5 - Protein expression and activity of cell cycle regulating proteins in parental and sunitinib-resistant 786-O cells after 48 h exposure to 1 or 1.5 µM SHI. Protein expression of Cyclin A (#), corresponding protein expression of ß-actin (*). n.r. = not relevant.

Figure S2

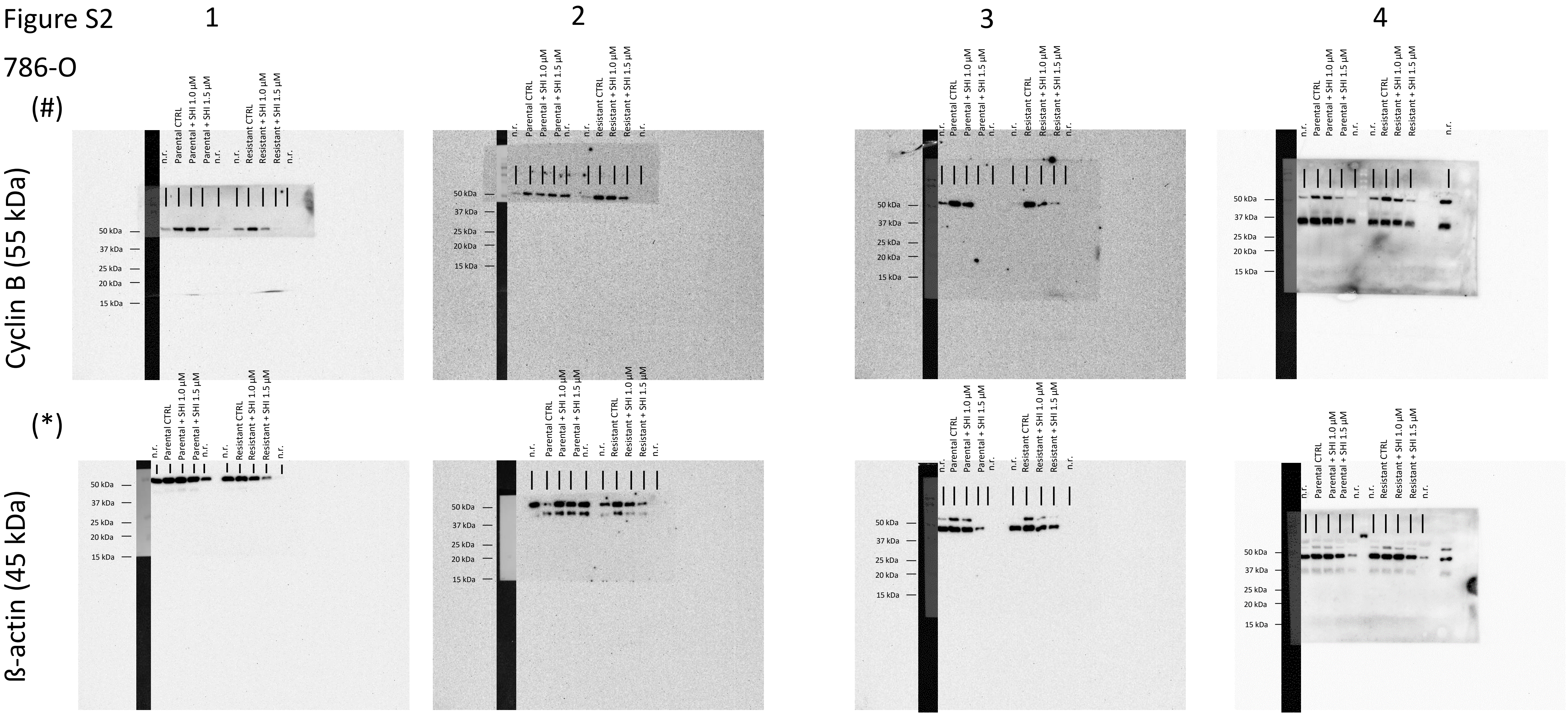

Figure S2d: Detailed information about Figure 5 - Protein expression and activity of cell cycle regulating proteins in parental and sunitinib-resistant 786-O cells after 48 h exposure to 1 or 1.5 µM SHI. Protein expression of Cyclin B (#), corresponding protein expression of ß-actin (*). n.r. = not relevant.

Figure S2

786-O

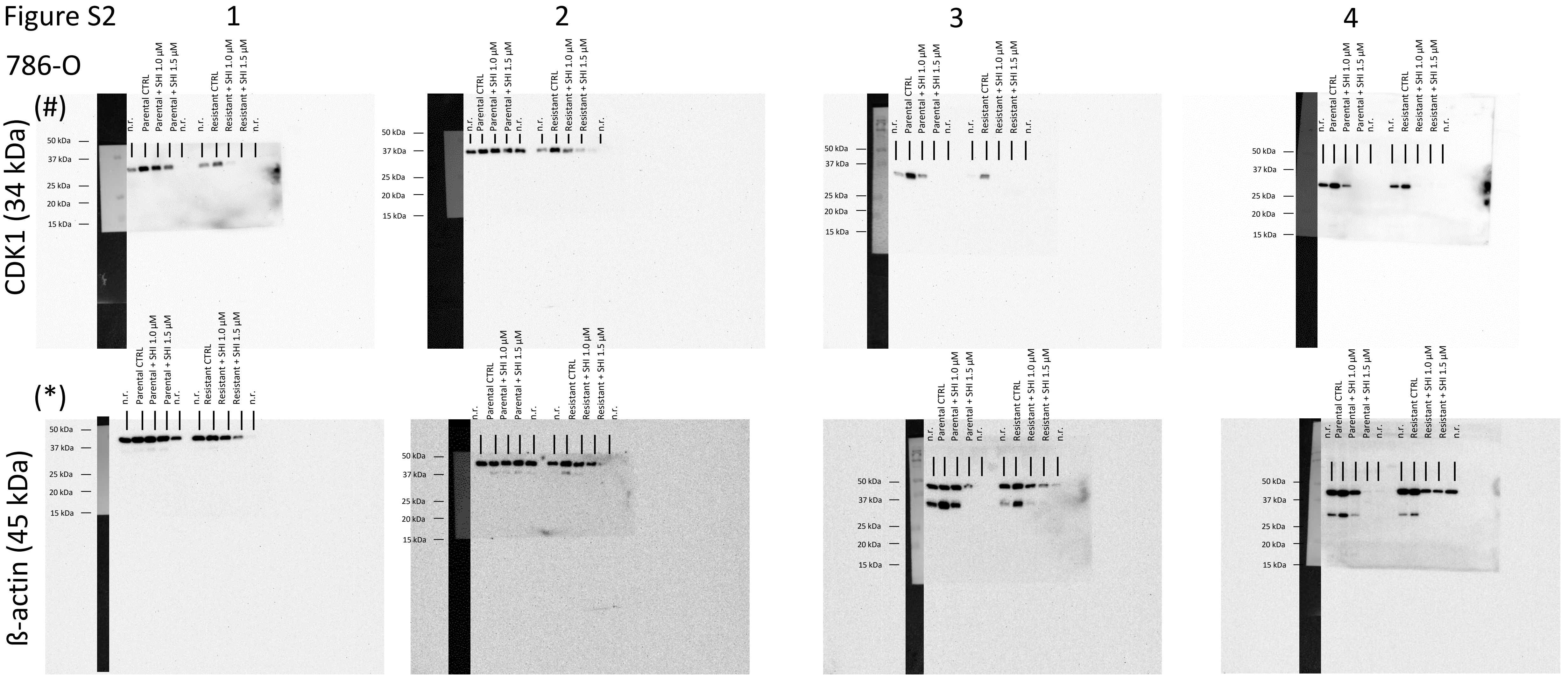

Figure S2e: Detailed information about Figure 5 - Protein expression and activity of cell cycle regulating proteins in parental and sunitinib-resistant 786-O cells after 48 h exposure to 1 or 1.5 µM SHI. Protein expression of CDK1 (#), corresponding protein expression of ß-actin (*). n.r. = not relevant.

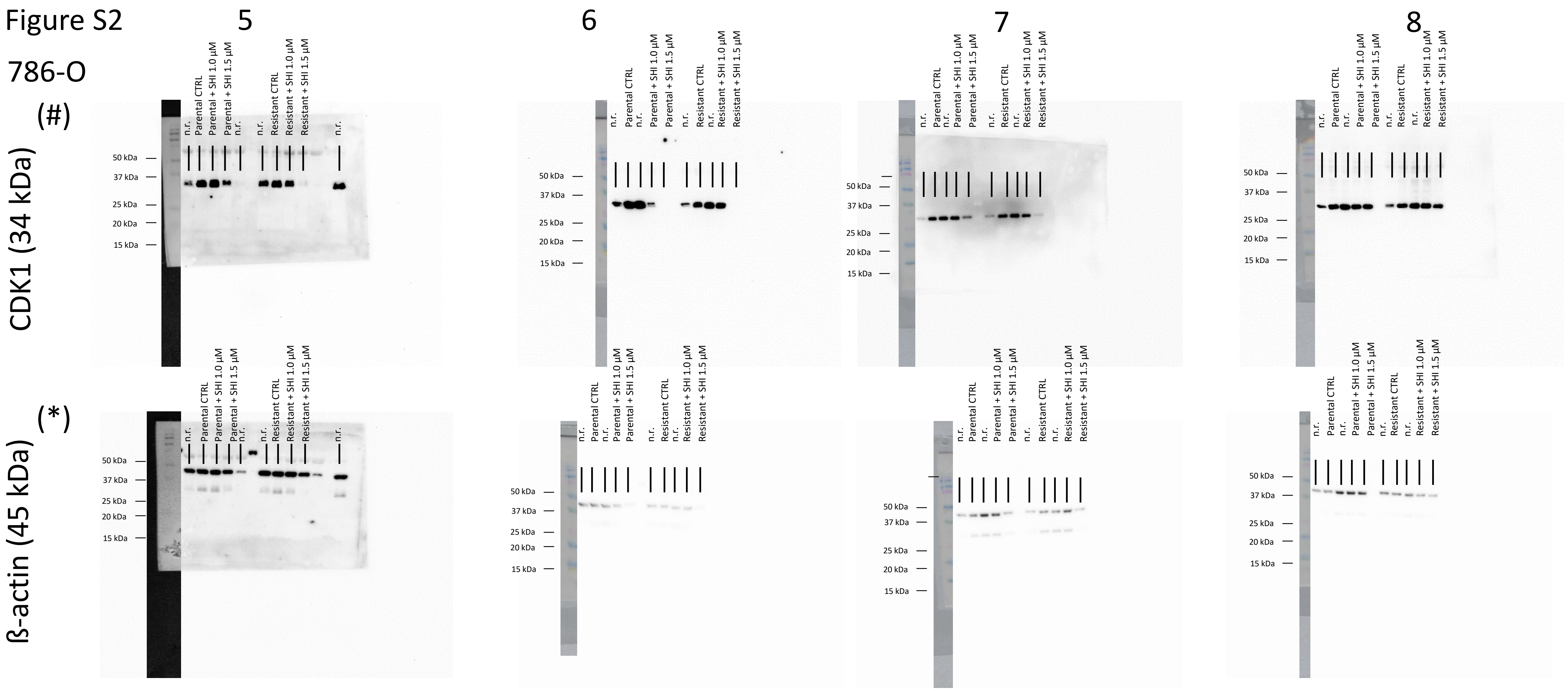

Figure S2e: Detailed information about Figure 5 - Protein expression and activity of cell cycle regulating proteins in parental and sunitinib-resistant 786-O cells after 48 h exposure to 1 or 1.5 µM SHI. Protein expression of CDK1 (#), corresponding protein expression of ß-actin (*). n.r. = not relevant.

Figure S2

786-O

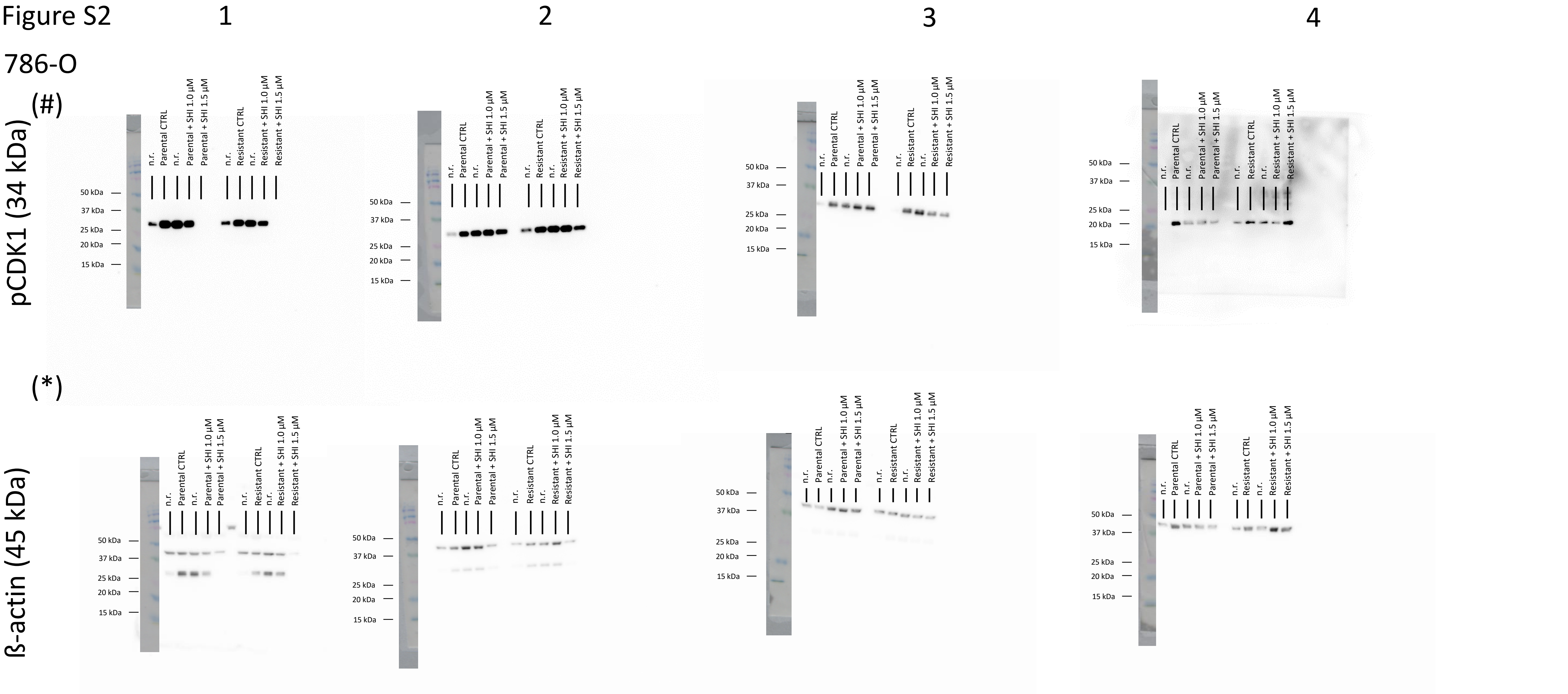

Figure S2f: Detailed information about Figure 5 - Protein expression and activity of cell cycle regulating proteins in parental and sunitinib-resistant 786-O cells after 48 h exposure to 1 or 1.5 µM SHI. Protein expression of pCDK1 (#), corresponding protein expression of ß-actin (*). n.r. = not relevant.

Figure S2

786-O

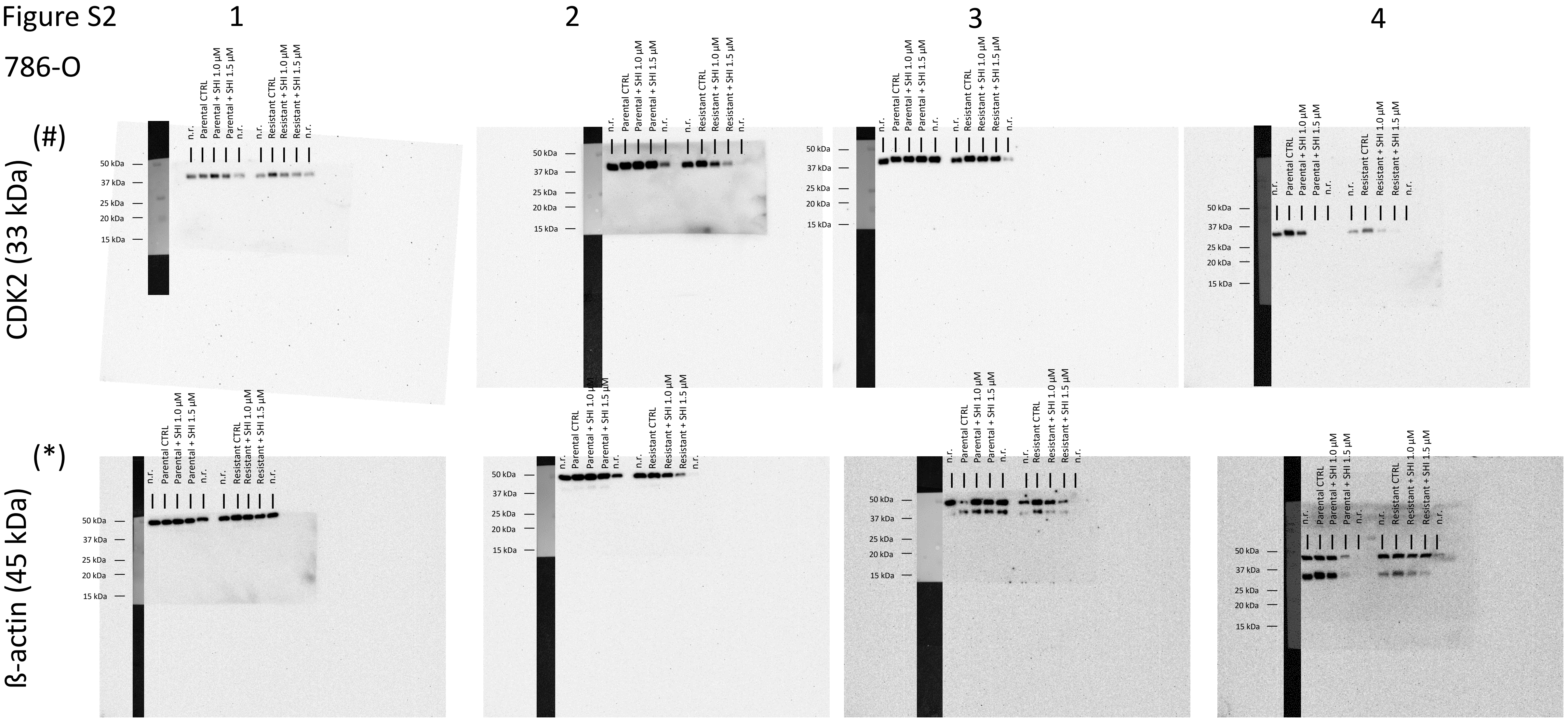

Figure S2g: Detailed information about Figure 5 - Protein expression and activity of cell cycle regulating proteins in parental and sunitinib-resistant 786-O cells after 48 h exposure to 1 or 1.5 µM SHI. Protein expression of CDK2 (#), corresponding protein expression of ß-actin (*). n.r. = not relevant.

Figure S2

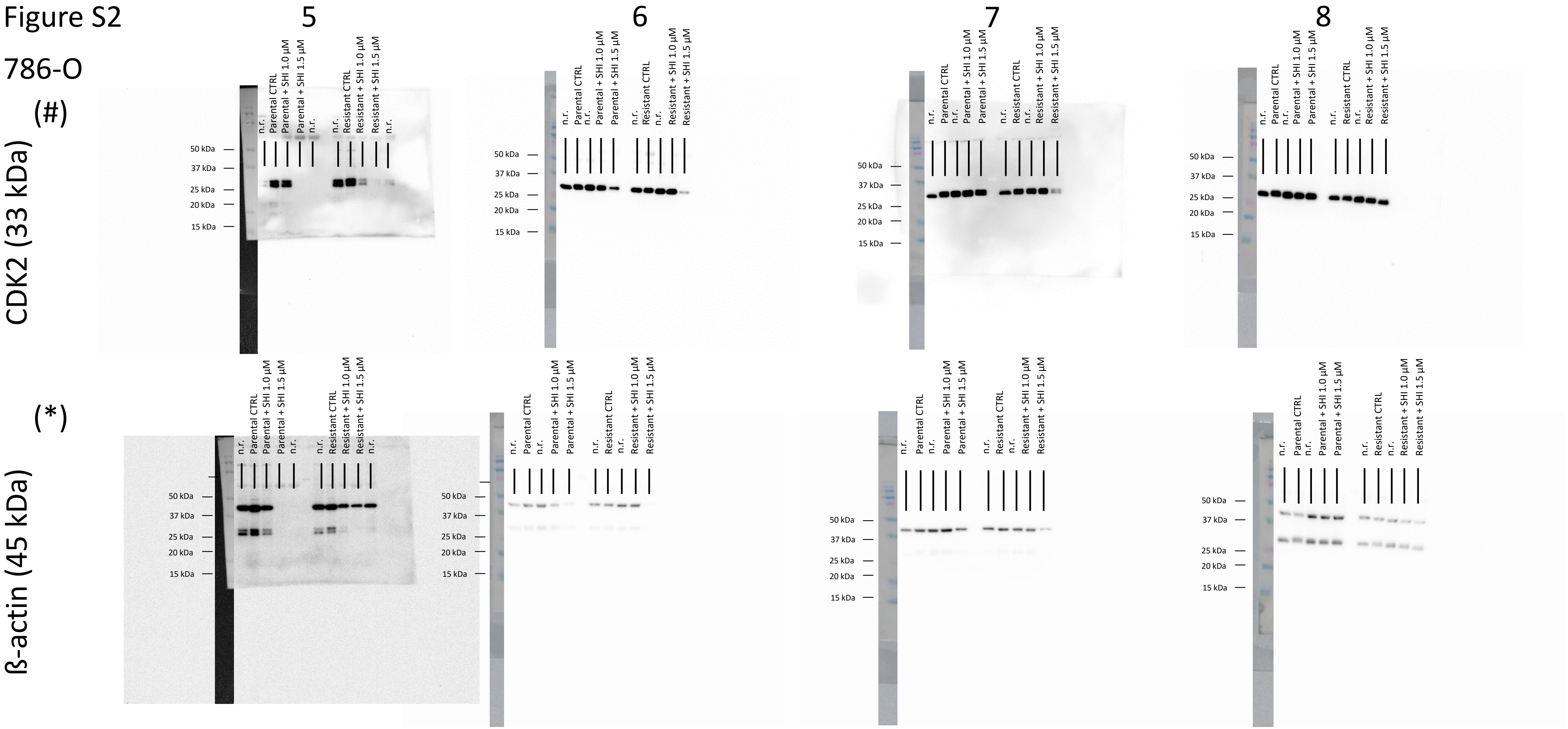

Figure S2g: Detailed information about Figure 5 - Protein expression and activity of cell cycle regulating proteins in parental and sunitinib-resistant 786-O cells after 48 h exposure to 1 or 1.5 µM SHI. Protein expression of CDK2 (#), corresponding protein expression of ß-actin (*). n.r. = not relevant.

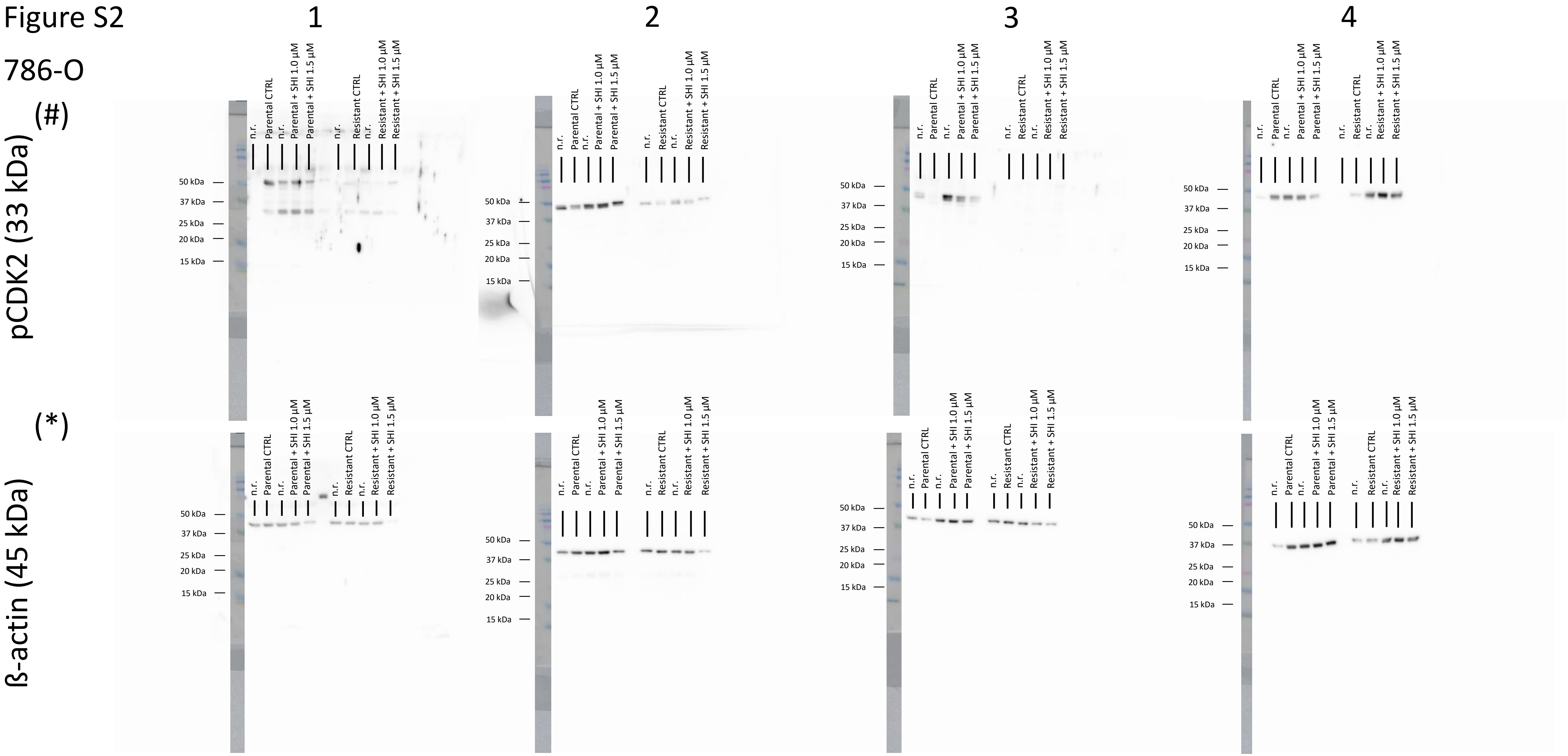

Figure S2f: Detailed information about Figure 5 - Protein expression and activity of cell cycle regulating proteins in parental and sunitinib-resistant 786-O cells after 48 h exposure to 1 or 1.5 µM SHI. Protein expression of pCDK2 (#), corresponding protein expression of ß-actin (*). n.r. = not relevant.

Nekroptose proteins

RIP1, pRIP1, RIP3, pRIP3, MLKL, pMLKL

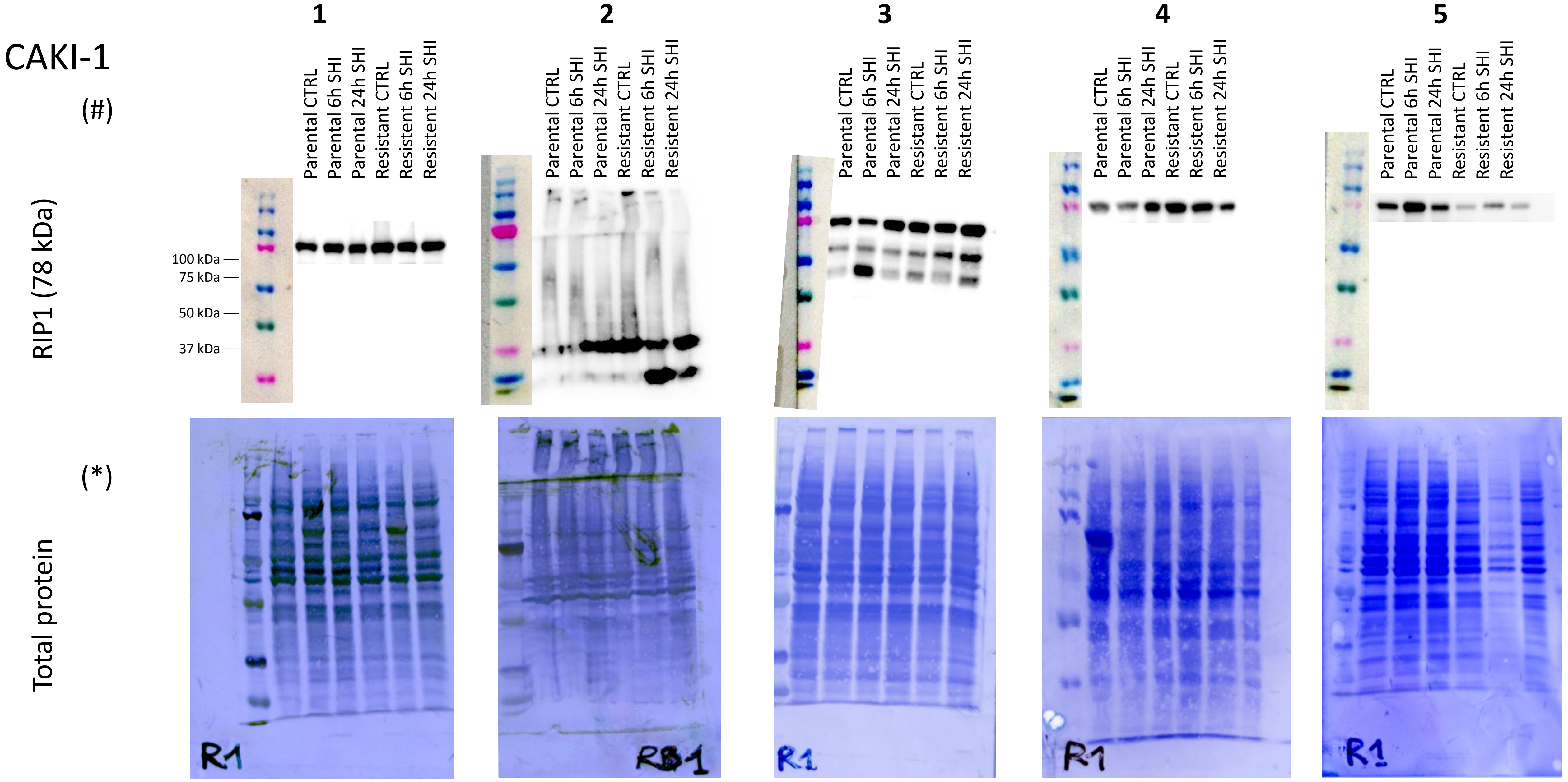

Figure S3a: Detailed information about Figure 10 - Necrosome complex key proteins in parental and sunitinib-resistant Caki-1 cells after 6 and 24 h exposure to 1 µM SHI. Protein expression of RIP1 (#), corresponding Coomassie blue staining of total protein (*).

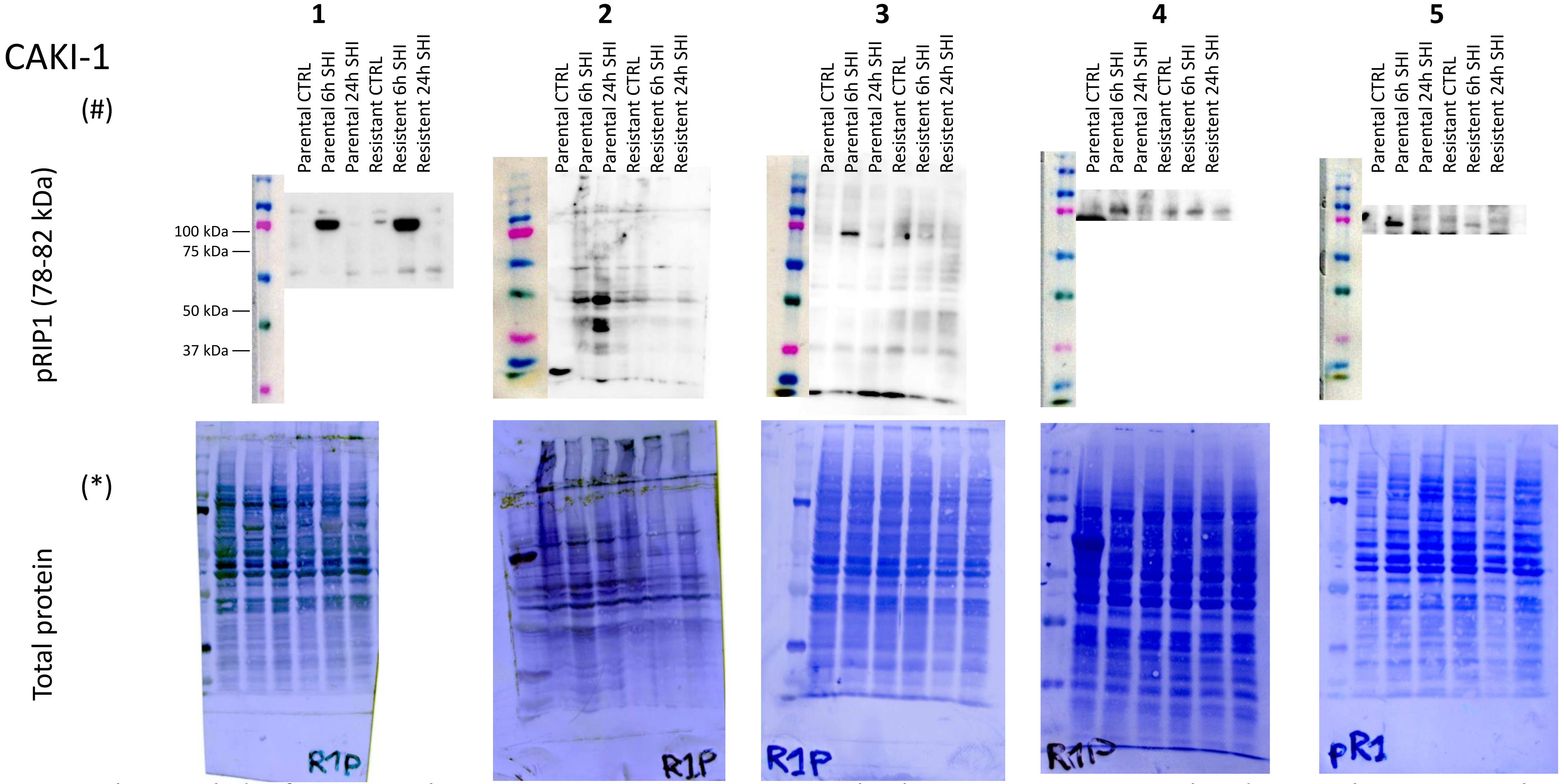

Figure S3b: Detailed information about Figure 10 - Necrosome complex key proteins in parental and sunitinib-resistant Caki-1 cells after 6 and 24 h exposure to 1 µM SHI. Protein expression of pRIP1 (#), corresponding Coomassie blue staining of total protein (*).

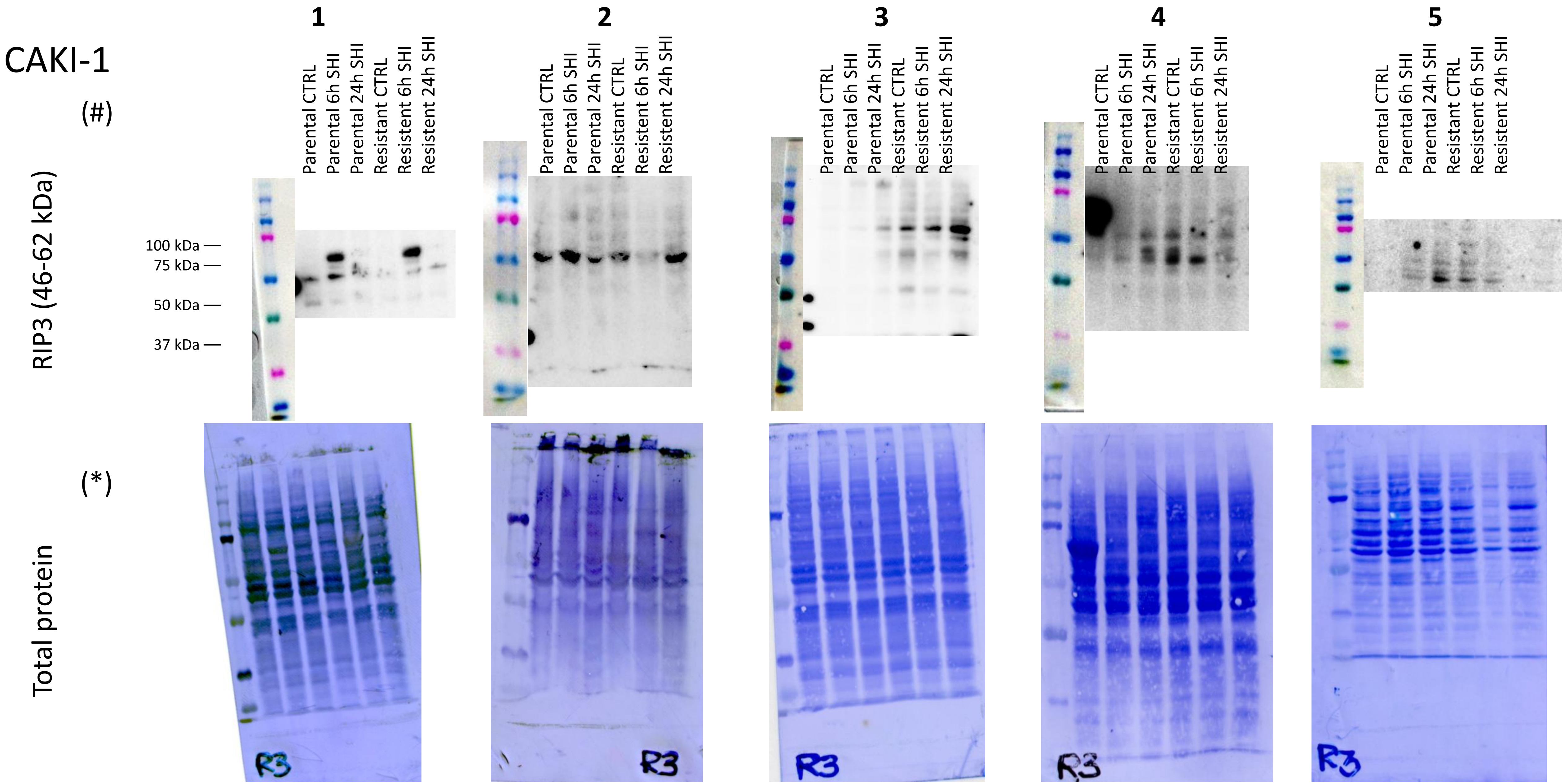

Figure S3c: Detailed information about Figure 10 - Necrosome complex key proteins in parental and sunitinib-resistant Caki-1 cells after 6 and 24 h exposure to 1 µM SHI. Protein expression of RIP3 (#), corresponding Coomassie blue staining of total protein (*).

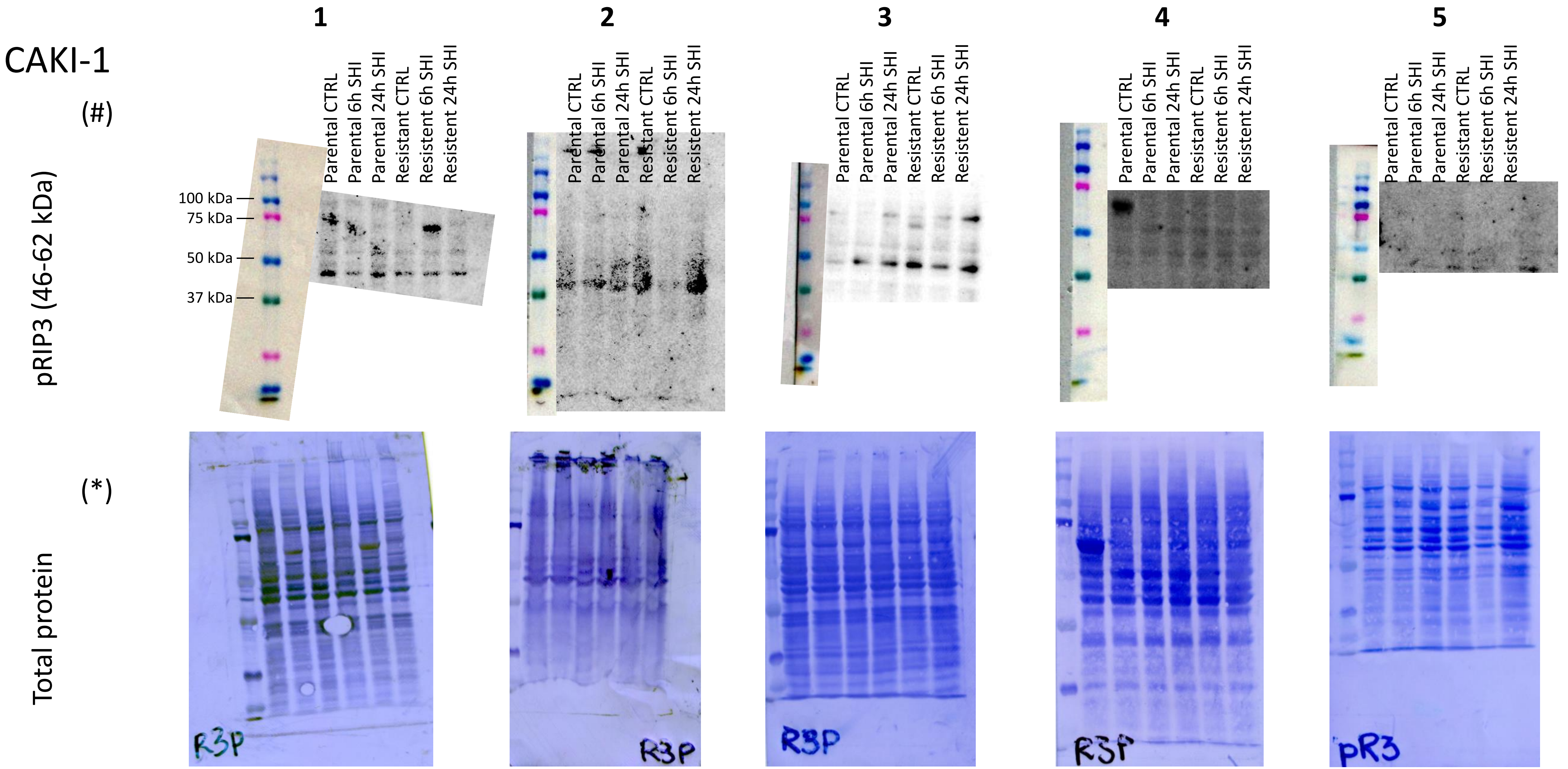

Figure S3d: Detailed information about Figure 10 - Necrosome complex key proteins in parental and sunitinib-resistant Caki-1 cells after 6 and 24 h exposure to 1 µM SHI. Protein expression of pRIP3 (#), corresponding Coomassie blue staining of total protein (*).

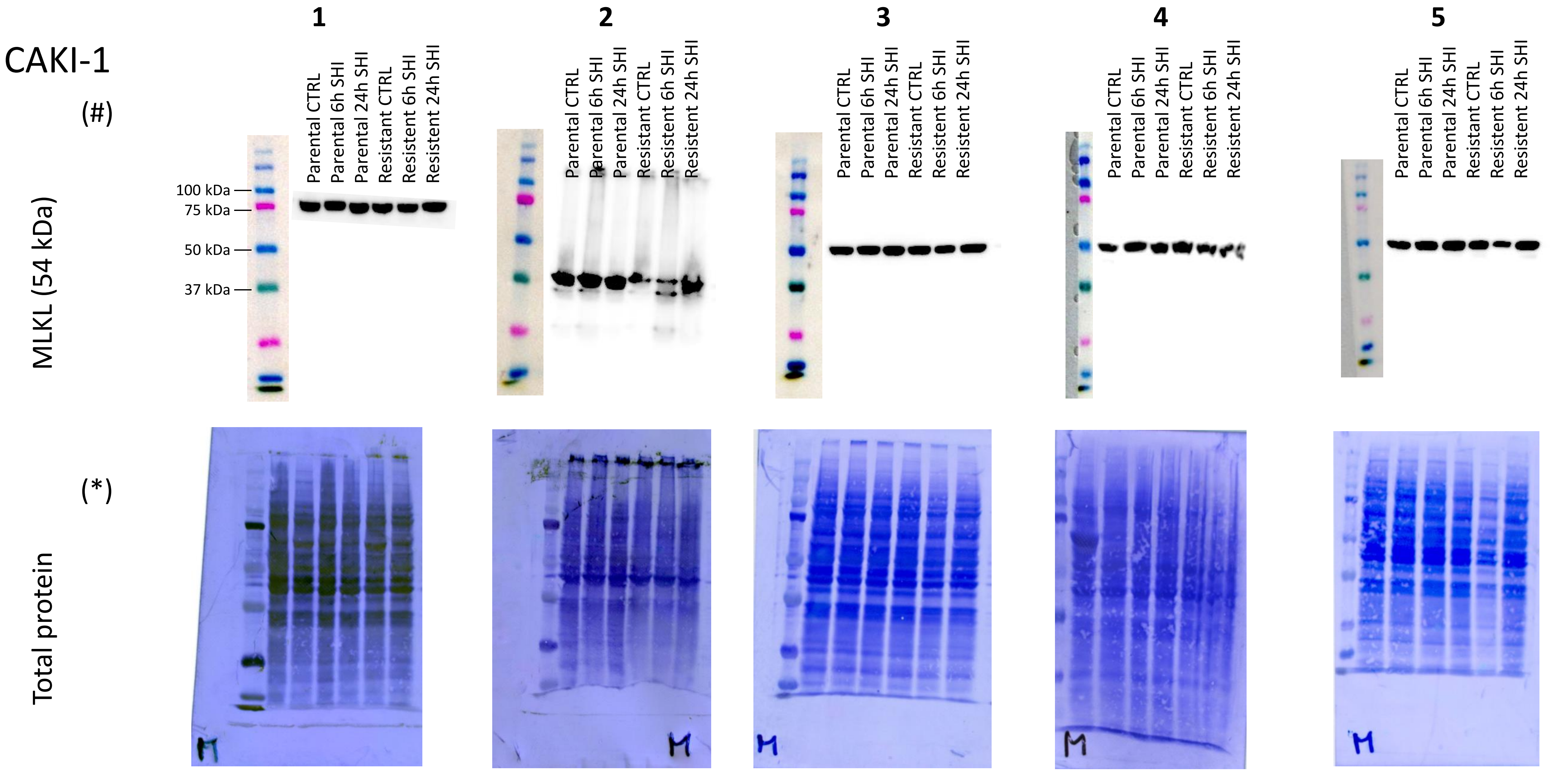

Figure S3e: Detailed information about Figure 10 - Necrosome complex key proteins in parental and sunitinib-resistant Caki-1 cells after 6 and 24 h exposure to 1 µM SHI. Protein expression of MLKL (#), corresponding Coomassie blue staining of total protein (*).

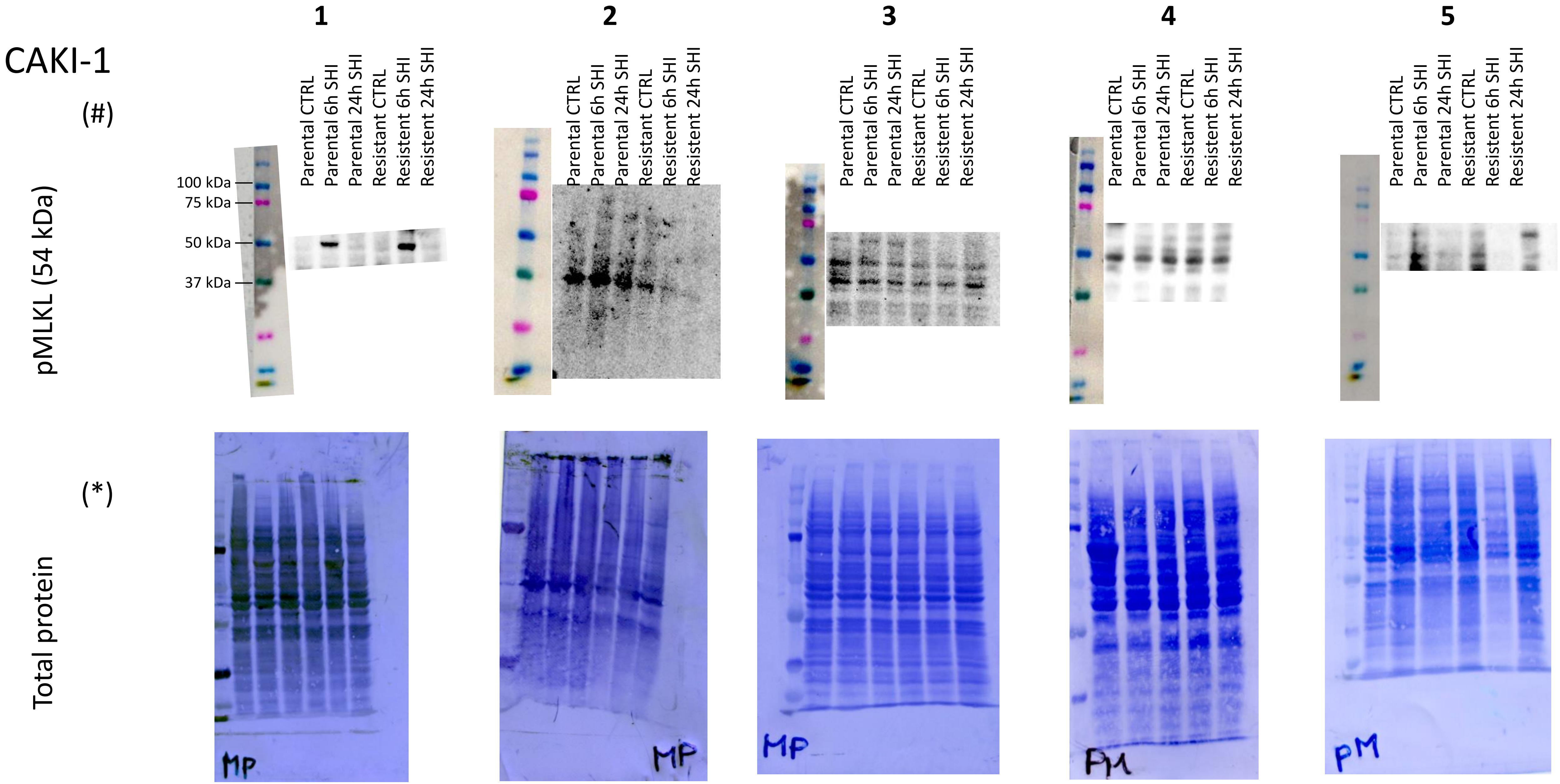

Figure S3f: Detailed information about Figure 10 - Necrosome complex key proteins in parental and sunitinib-resistant Caki-1 cells after 6 and 24 h exposure to 1 µM SHI. Protein expression of pMLKL (#), corresponding Coomassie blue staining of total protein (*).

Signal proteins

AKT, pAKT, mTOR, pmTOR

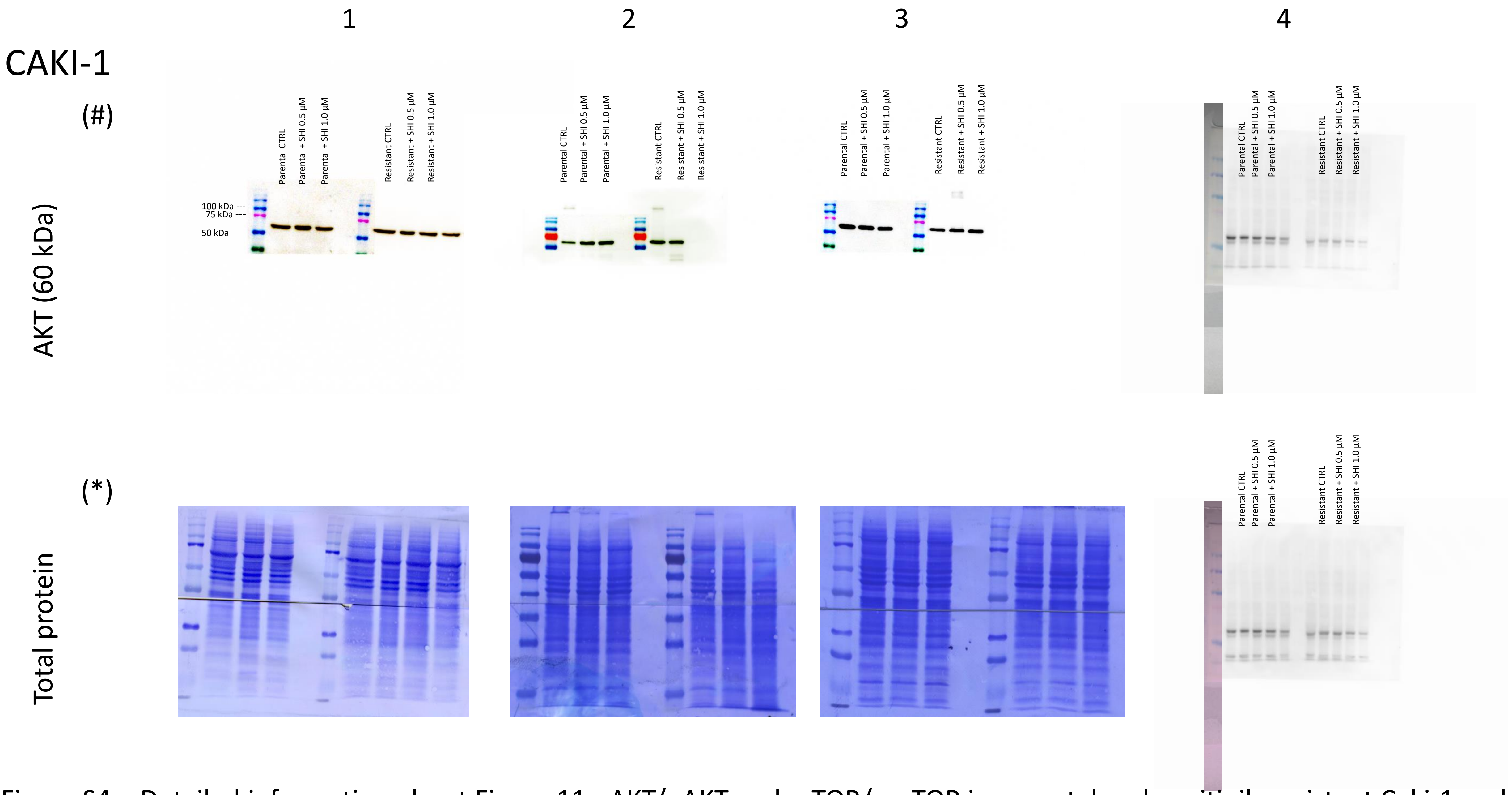

Figure S4a: Detailed information about Figure 11 - AKT/pAKT and mTOR/pmTOR in parental and sunitinib-resistant Caki-1 and 786-O cells after 48 h exposure to SHI [0.5, 1.0 µM]. Protein expression of AKT (#), corresponding protein expression of ß-actin or Coomassie blue staining of total protein (*).

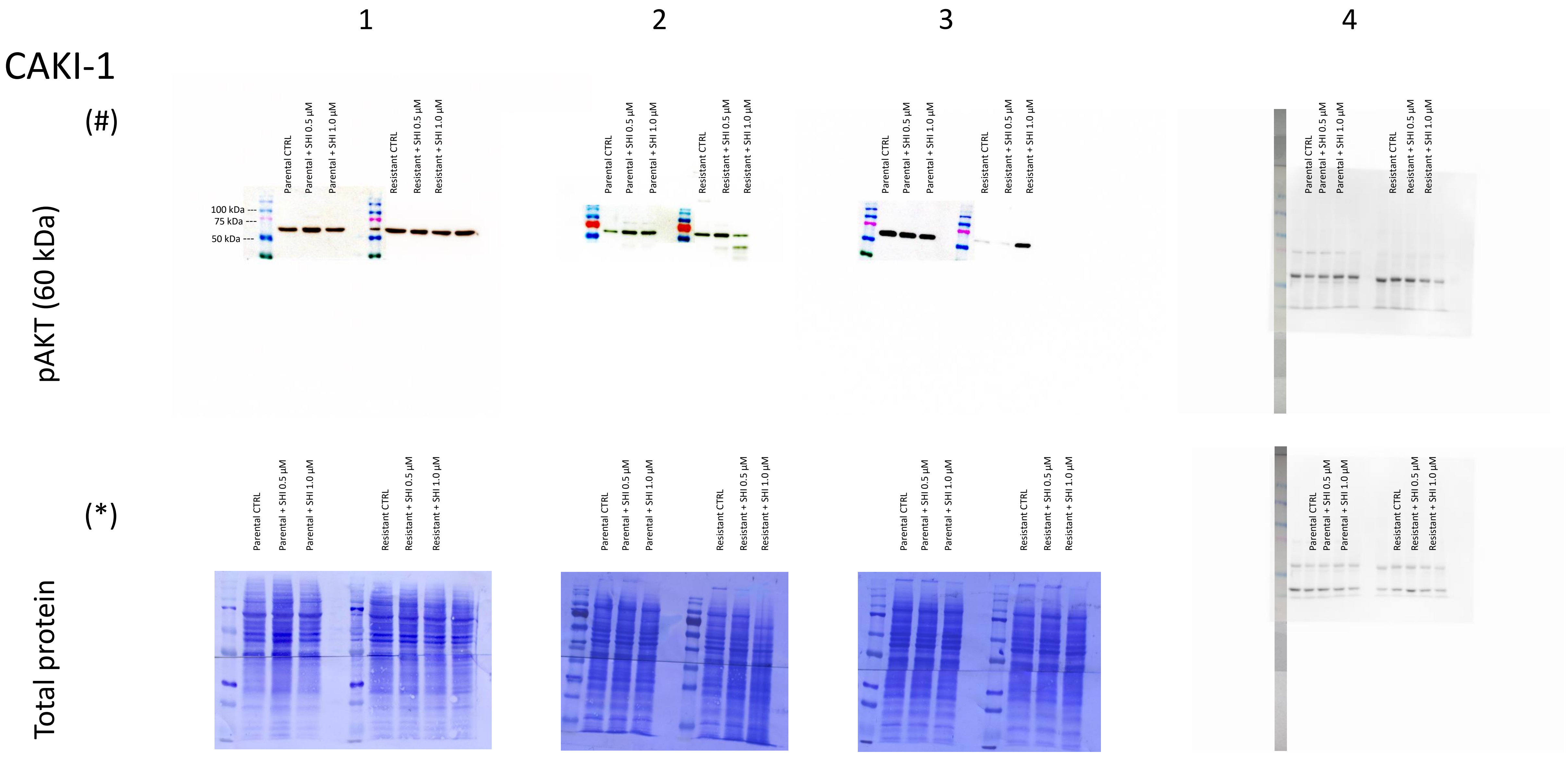

Figure S4b: Detailed information about Figure 11 - AKT/pAKT and mTOR/pmTOR in parental and sunitinib-resistant Caki-1 and 786-O cells after 48 h exposure to SHI [0.5, 1.0 µM]. Protein expression of pAKT (#), corresponding protein expression of ß-actin or Coomassie blue staining of total protein (*).

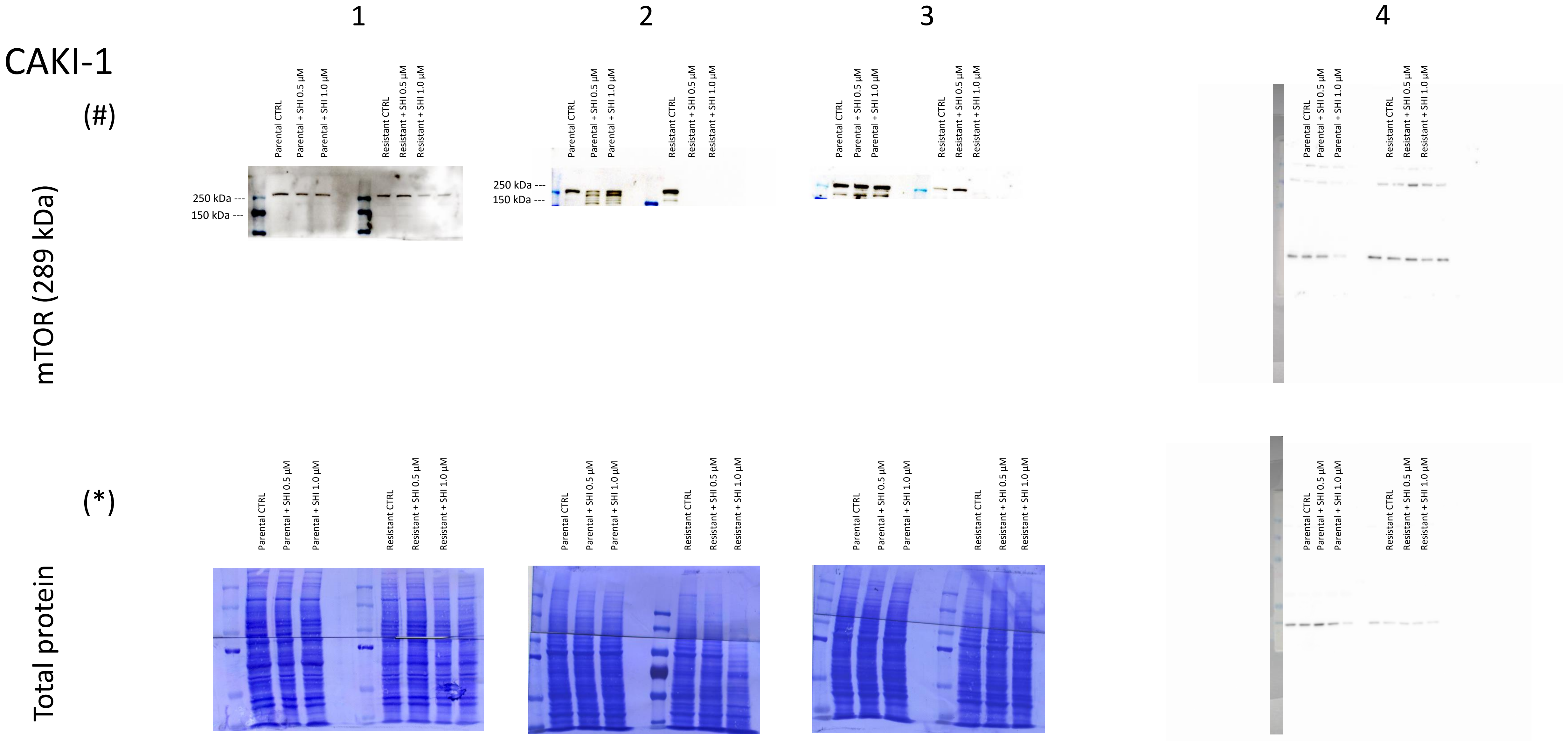

Figure S4c: Detailed information about Figure 11 - AKT/pAKT and mTOR/pmTOR in parental and sunitinib-resistant Caki-1 and 786-O cells after 48 h exposure to SHI [0.5, 1.0 µM]. Protein expression of mTOR (#), corresponding protein expression of ß-actin or Coomassie blue staining of total protein (*).

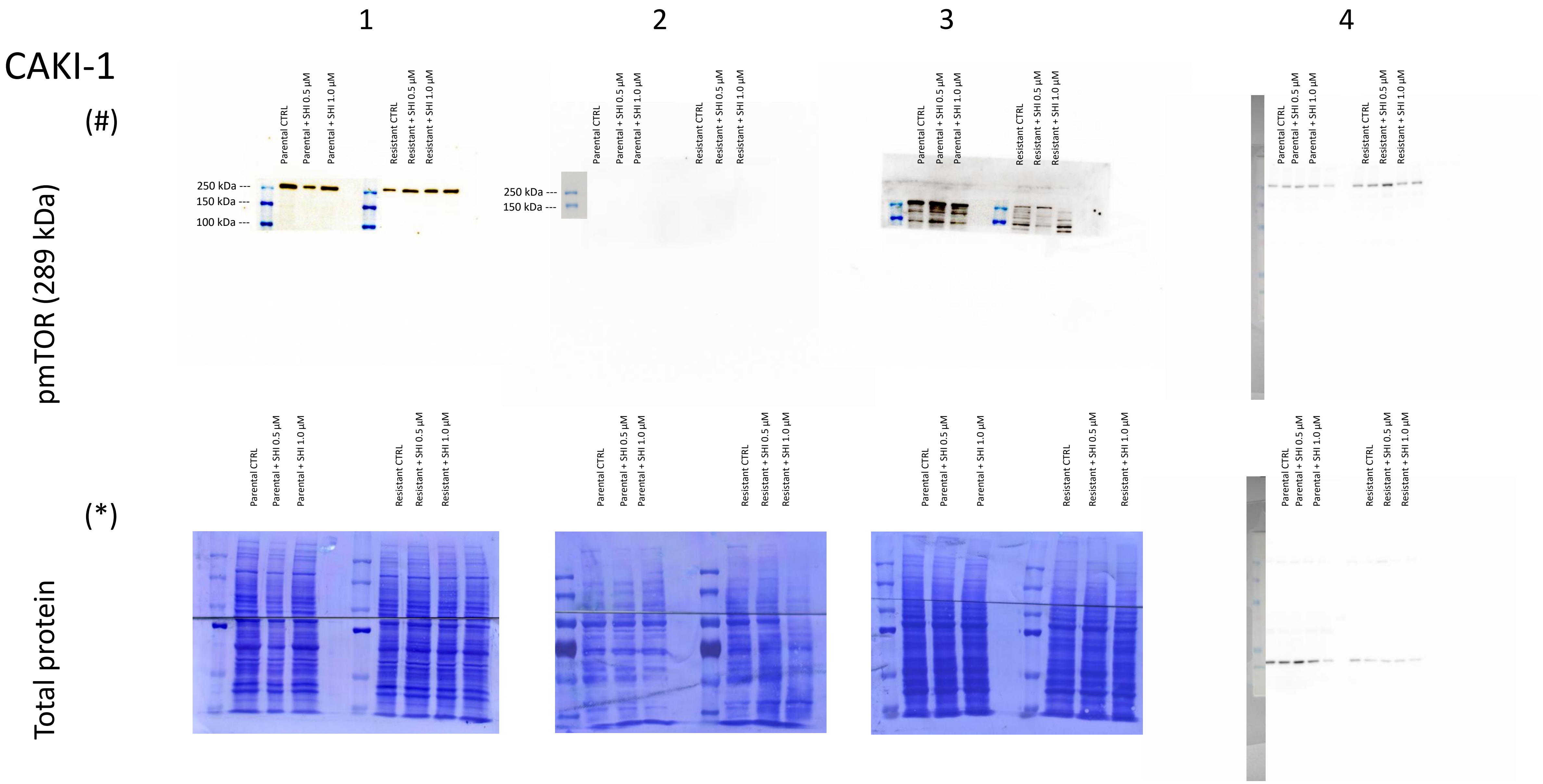

Figure S4d: Detailed information about Figure 11 - AKT/pAKT and mTOR/pmTOR in parental and sunitinib-resistant Caki-1 and 786-O cells after 48 h exposure to SHI [0.5, 1.0 µM]. Protein expression of pmTOR (#), corresponding protein expression of ß-actin or Coomassie blue staining of total protein (*).

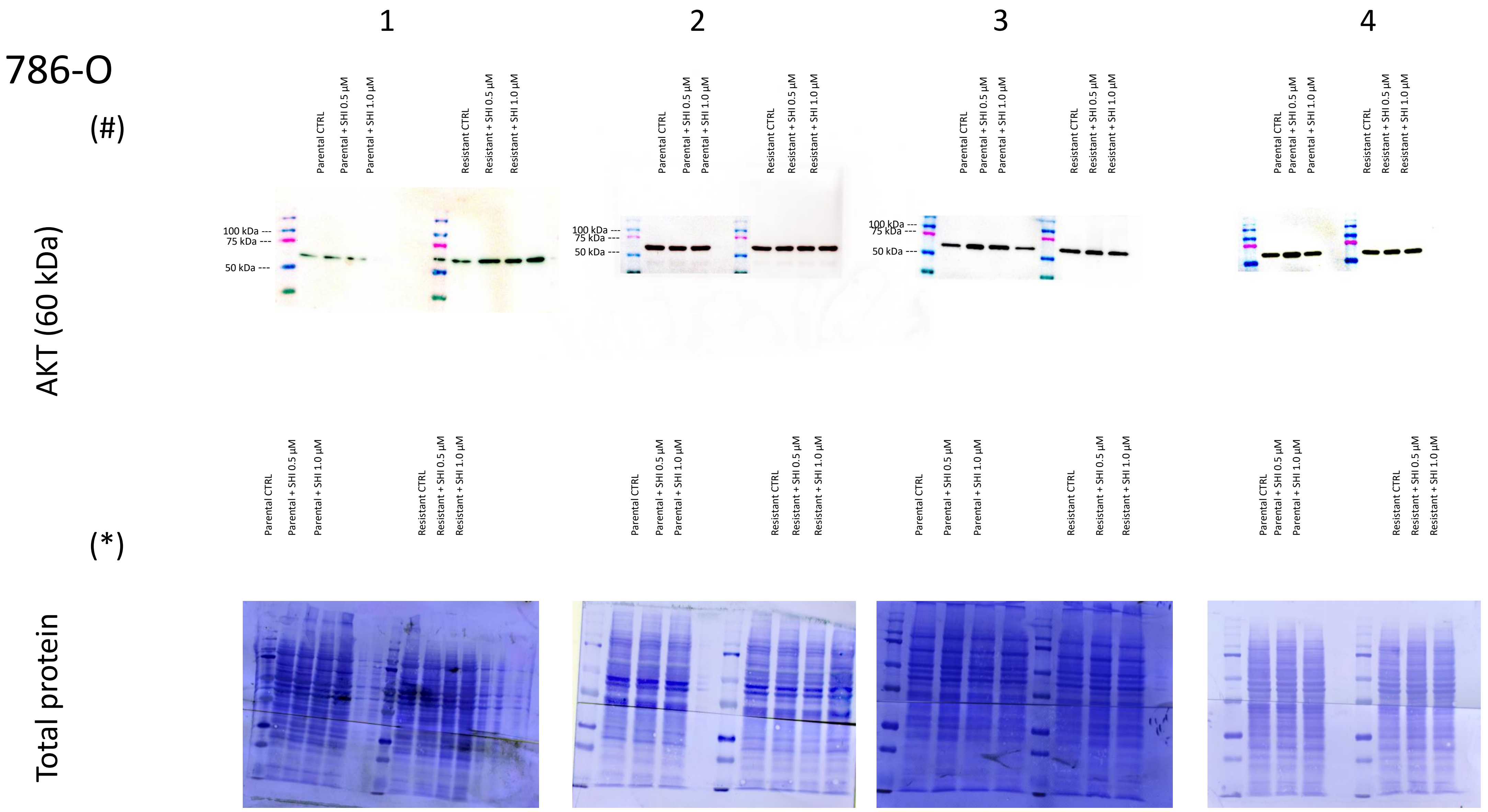

Figure S4e: Detailed information about Figure 11 - AKT/pAKT and mTOR/pmTOR in parental and sunitinib-resistant Caki-1 and 786-O cells after 48 h exposure to SHI [0.5, 1.0 µM]. Protein expression of AKT (#), corresponding Coomassie blue staining of total protein (*).

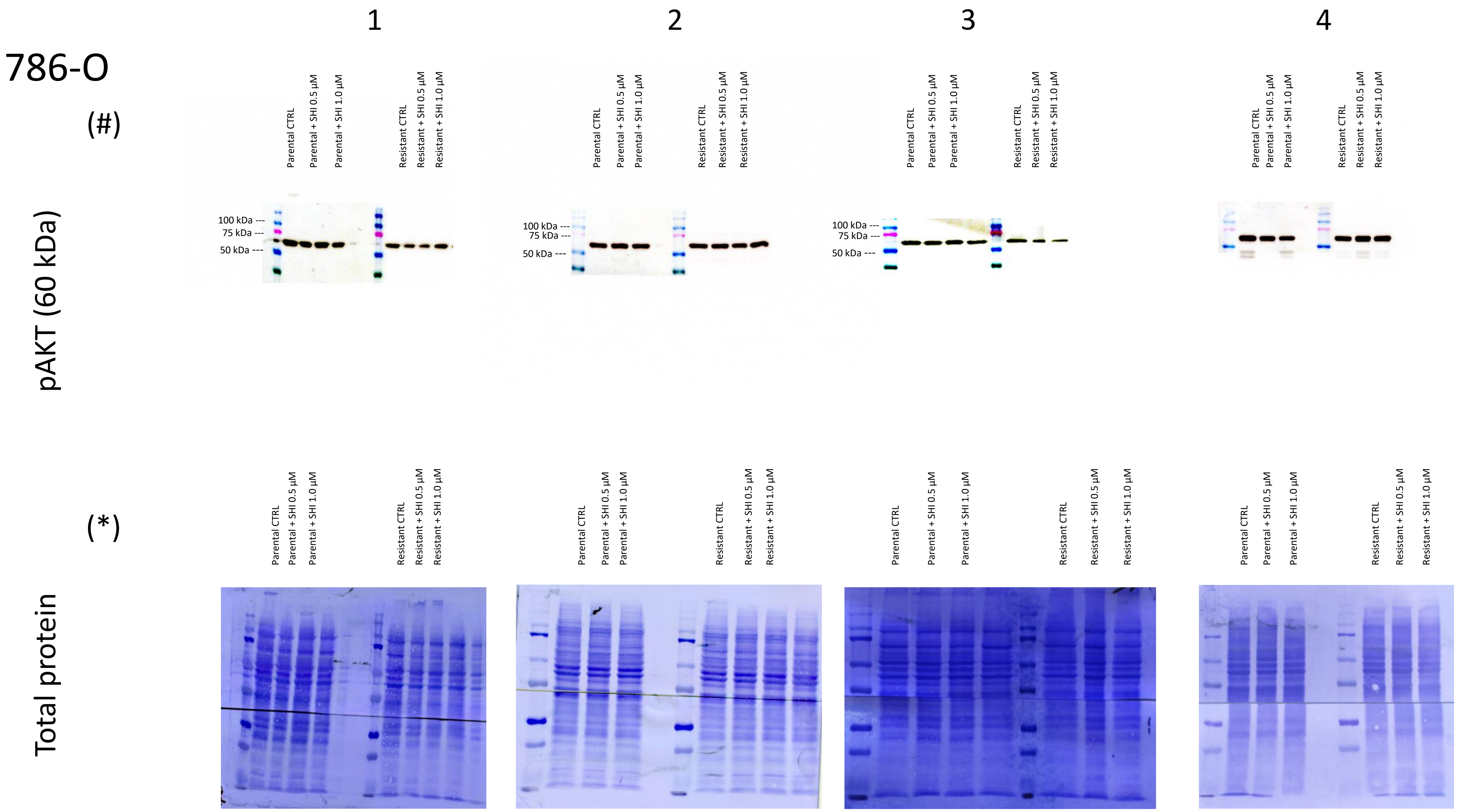

Figure S4f: Detailed information about Figure 11 - AKT/pAKT and mTOR/pmTOR in parental and sunitinib-resistant Caki-1 and 786-O cells after 48 h exposure to SHI [0.5, 1.0 µM]. Protein expression of pAKT (#), corresponding Coomassie blue staining of total protein (*).

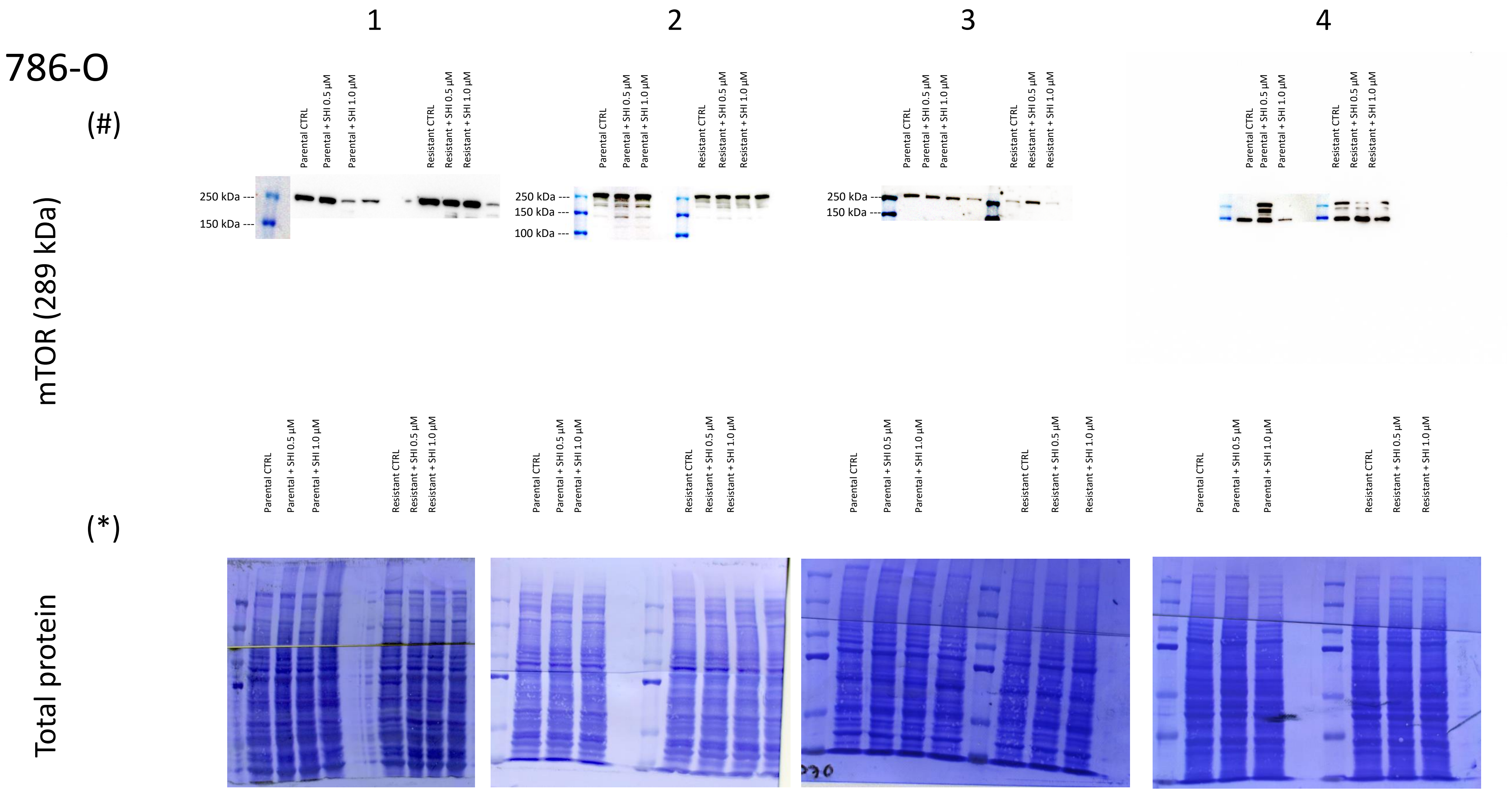

Figure S4g: Detailed information about Figure 11 - AKT/pAKT and mTOR/pmTOR in parental and sunitinib-resistant Caki-1 and 786-O cells after 48 h exposure to SHI [0.5, 1.0 µM]. Protein expression of mTOR (#), corresponding Coomassie blue staining of total protein (*).

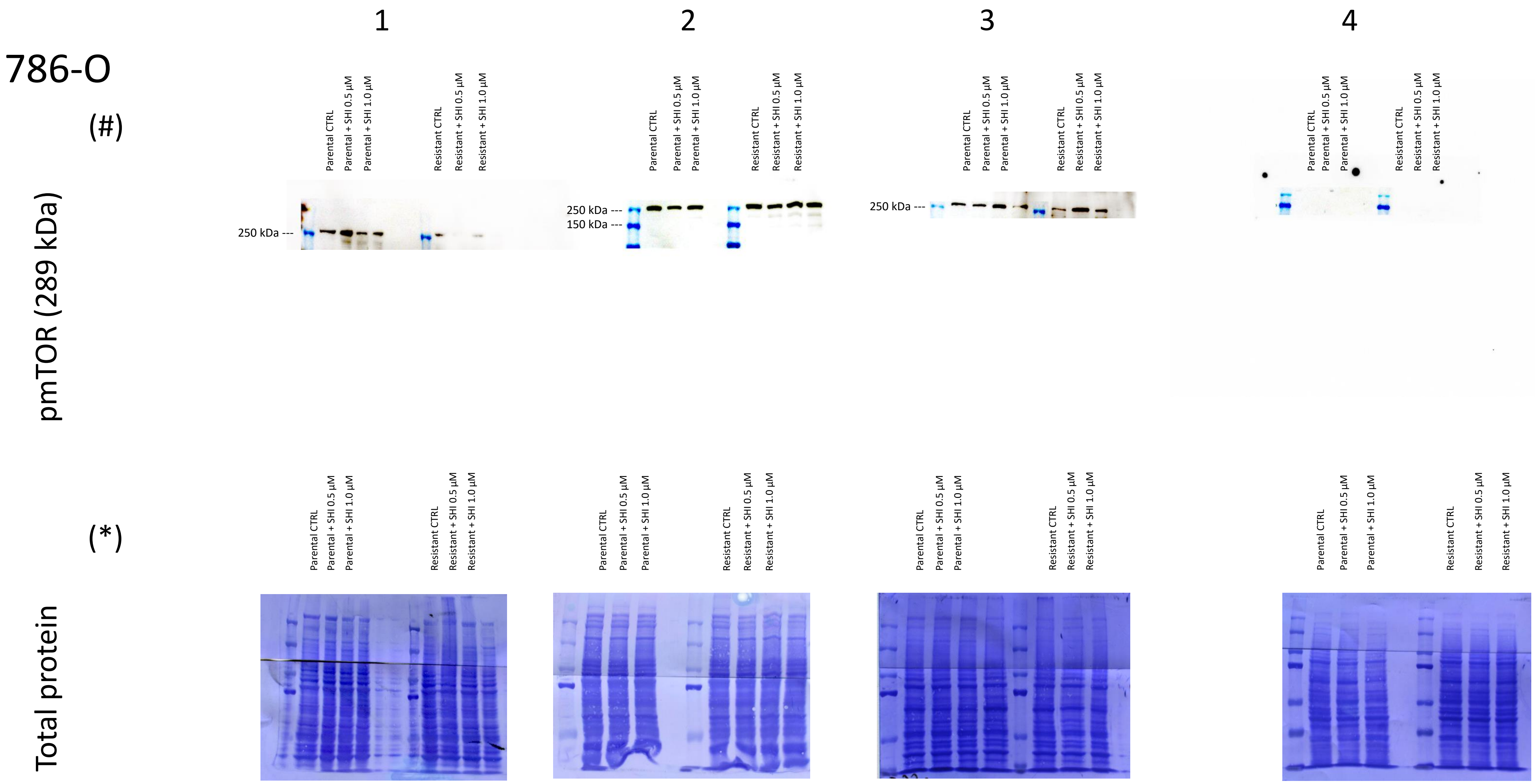

Figure S4h: Detailed information about Figure 11 - AKT/pAKT and mTOR/pmTOR in parental and sunitinib-resistant Caki-1 and 786-O cells after 48 h exposure to SHI [0.5, 1.0 µM]. Protein expression of pmTOR (#), corresponding Coomassie blue staining of total protein (*).
